# Cross-species conservation of episome maintenance provides a basis for in vivo investigation of Kaposi's sarcoma herpesvirus LANA

**DOI:** 10.1371/journal.ppat.1006555

**Published:** 2017-09-14

**Authors:** Aline C. Habison, Marta Pires de Miranda, Chantal Beauchemin, Min Tan, Sofia A. Cerqueira, Bruno Correia, Rajesh Ponnusamy, Edward J. Usherwood, Colin E. McVey, J. Pedro Simas, Kenneth M. Kaye

**Affiliations:** 1 Departments of Medicine, Brigham and Women’s Hospital, Harvard Medical School, Boston, Massachusetts, United States of America; 2 Instituto de Medicina Molecular, Faculdade de Medicina, Universidade de Lisboa, Lisboa, Portugal; 3 Instituto de Tecnologia Quimica e Bioliogica Antonio Xavier, Universidade Nova de Lisboa, Oeiras, Portugal; 4 Department of Microbiology and Immunology, Geisel School of Medicine at Dartmouth, Lebanon, New Hampshire, United States of America; University of Utah, UNITED STATES

## Abstract

Many pathogens, including Kaposi’s sarcoma herpesvirus (KSHV), lack tractable small animal models. KSHV persists as a multi-copy, nuclear episome in latently infected cells. KSHV latency-associated nuclear antigen (kLANA) binds viral terminal repeat (kTR) DNA to mediate episome persistence. Model pathogen murine gammaherpesvirus 68 (MHV68) mLANA acts analogously on mTR DNA. kLANA and mLANA differ substantially in size and kTR and mTR show little sequence conservation. Here, we find kLANA and mLANA act reciprocally to mediate episome persistence of TR DNA. Further, kLANA rescued mLANA deficient MHV68, enabling a chimeric virus to establish latent infection in vivo in germinal center B cells. The level of chimeric virus in vivo latency was moderately reduced compared to WT infection, but WT or chimeric MHV68 infected cells had similar viral genome copy numbers as assessed by immunofluorescence of LANA intranuclear dots or qPCR. Thus, despite more than 60 Ma of evolutionary divergence, mLANA and kLANA act reciprocally on TR DNA, and kLANA functionally substitutes for mLANA, allowing kLANA investigation in vivo. Analogous chimeras may allow in vivo investigation of genes of other human pathogens.

## Author summary

KSHV latently infects cells and persists as a multi-copy, nuclear episome. KSHV LANA (kLANA) maintains episomes by acting on viral terminal repeat (kTR) elements. Model pathogen MHV68 mLANA acts analogously on mTR DNA. To date, KSHV investigation has been limited by lack of a tractable, small animal model. Here, we find that despite 60 Ma of evolutionary divergence, kLANA and mLANA exhibit inter-species functionality, acting reciprocally on TR DNA to mediate episome persistence. Further, kLANA rescued mLANA deficient MHV68, allowing chimeric virus to establish latent infection in vivo. The level of in vivo latency was moderately lower for kLANA chimeric virus compared to that of WT, but chimeric and WT virus infected cells had similar virus genome copy numbers. These results now provide a tractable model to investigate kLANA in vivo. This chimeric approach has the potential to be broadly applied to other small animal models for human pathogens.

## Introduction

Kaposi’s sarcoma-associated herpesvirus (KSHV), a gamma-2 herpesvirus, is the etiologic agent of Kaposi’s sarcoma, primary effusion lymphoma, and multicentric Castleman’s disease[1–5]. KSHV infection of tumor cells is predominantly latent. During latent infection, KSHV persists as a nuclear, multi-copy, extrachromosomal, circular episome[6]. To persist in proliferating cells, genomes must replicate with each cell division and segregate to progeny nuclei.

**Fig 1:**
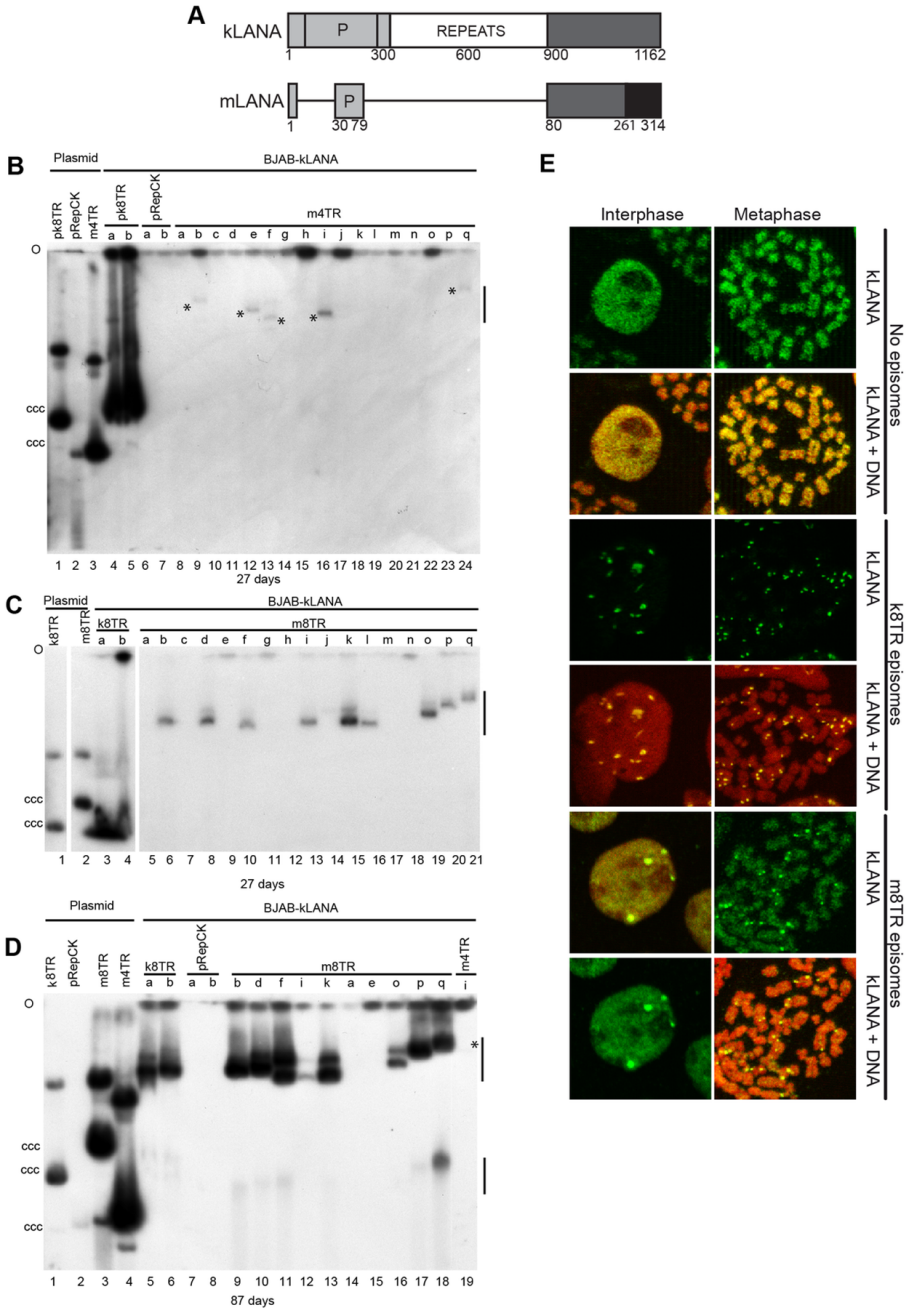
kLANA mediates mTR episome persistence. (A) Schematic of kLANA and mLANA. Homologous regions are indicated in grey shading. White and black regions share no homology. Amino acid residue numbers are indicated. P, proline-rich. Gardella gels after transfection of m4TR (B) or m8TR (C) DNA. Blots in B and C were probed with ^32^P-pRepCK DNA. (D) Gardella gel after 87 days of indicated cell lines from panels B and C. Blot was probed with ^32^P-m8TR DNA. Days of G418 selection are below each panel. O, gel origin; ccc plasmid DNA is indicated. Lanes contain 1.5-2x10^6^ cells. Vertical lines at right (panels B, C, E) indicate positions of episomal bands. Asterisks indicate faint episomal bands. (E) Immune fluorescence for kLANA. m8TR cells are from cell line d (panels C, D). Brightness and contrast were uniformly adjusted in panels from the same field and red signal was uniformly enhanced for k8TR panels using Adobe Photoshop. Magnification, 630x.

The latency-associated nuclear antigen (kLANA) (Fig 1A) is one of a small subset of KSHV genes expressed in latency. LANA acts on KSHV terminal repeat (kTR) DNA to mediate episome persistence[7,8], for which it is essential[9]. N-terminal LANA binds histones H2A/H2B on the nucleosome surface to attach to mitotic chromosomes[10], and C-terminal LANA DNA binding domain (DBD) simultaneously binds adjacent LANA binding sites (LBSs) within TR DNA, to form a molecular tethering apparatus which ensures genomes are segregated to daughter cell nuclei following mitosis[8,11–14]. LANA also mediates KSHV DNA replication and exerts important transcriptional and growth effects[11,15–25].

Although KSHV infection is naturally limited to humans, the pursuit of a small animal model of infection to study pathogenesis has been a longstanding goal in the field and has been accomplished in several models of immune compromised mice. KSHV injection into SCID mice implanted with human fetal thymus and liver grafts led to lytic and latent infection with B cells most commonly infected[26], while injection into NOD/SCID mice resulted in latent and lytic gene expression over several months with infection of multiple cell types including B cells[27]. Oral, intraperitoneal or vaginal inoculation into a humanized NOD/SCID/IL2rgamma mouse implanted with human fetal liver and thymus permitted KSHV infection of B cells and macrophages[28]. These models are advantageous in allowing direct investigation of KSHV, but are limited by their requirement for immune suppressed mice.

Murine gamma-2 herpesvirus 68 (MHV68 or murid herpesvirus 4) infection of laboratory mice provides a complementary, well-characterized model for gammaherpesvirus investigation. After intranasal inoculation, MHV68 undergoes lytic infection in the lungs, disseminates to lymphoid organs where it establishes latency in the spleen, and drives proliferation of germinal center (GC) B cells[29–31]. MHV68 shares sequence homology and has a genome that is generally colinear with KSHV[32]. MHV68 encodes a LANA homolog (mLANA), which is smaller than kLANA but has a conserved C-terminal DNA binding domain (Fig 1A). Analogous to kLANA, mLANA acts on mTR DNA to mediate episome persistence[33]. Consistent with its central role in episome persistence, mLANA is essential for efficient establishment of MHV68 latency in vivo[34–36].

In this work, we investigated the possibility of inter-species functionality between MHV68 and KSHV LANA for episome maintenance. We found that kLANA can support episome persistence of plasmids containing mTR elements and similarly, mLANA can support episome persistence of kTR plasmids. Further, we found that a chimeric MHV68, expressing kLANA, but not mLANA, was capable of establishing latent infection in splenic GC B cells, thus providing a model for kLANA investigation in vivo.

## Results

### KSHV LANA acts on mTRs to establish episome persistence

Since KSHV and MHV68 LANAs share homology (Fig 1A), we assessed if kLANA can mediate episome persistence of MHV68 TR (mTR) elements. We transfected DNA (encoding G418 resistance) containing 8 (m8TR), 4 (m4TR), or 2 (m2TR) MHV68 TR copies, 8 KSHV TR (pk8TR) copies, or vector (pRepCK) into human BJAB B lymphoma cells stably expressing kLANA (BJAB-kLANA). k8TR exhibited much higher outgrowth from kLANA expressing cells as compared with control BJAB cells (S1 Table). The higher outgrowth is due to the much higher efficiency of kLANA mediated episome persistence compared with integration. Similarly, m8TR, m4TR, and m2TR G418 resistant outgrowth was also increased in BJAB-kLANA cells compared with BJAB cells, consistent with possible kLANA mediated episome persistence.

G148 resistant cell lines were expanded and assessed for the presence of episomes by Gardella gels. In Gardella gels, live cells are loaded into gel wells, and lysed *in situ*. During electrophoresis, chromosomal DNA remains at the gel origin, whereas episomal DNA as large as several hundred kilobases migrates into the gel. As expected, BJAB-kLANA cells transfected with pk8TR showed strong episomal signal in all tested cell lines (Fig 1B–1D)(Table 1). Also, as expected, BJAB-kLANA cells transfected with vector pRepCK, which does not contain TR sequence, lacked episomes (Fig 1B and 1D). Transfection of m2TR DNA into BJAB-kLANA cells resulted in only one of 31 (3%) G418 resistant cell lines with episomes (Table 1). After transfection of m4TR DNA into BJAB-kLANA cells and G418 selection for 27 days, episomes were detected in five (Fig 1B, (indicated with asterisks)) of 17 lanes, and in three experiments, 12 of 37 (32%) G418 resistant cell lines contained episomes. The episomal signal was substantially weaker for the m4TR episomes compared to the pk8TR episomal DNA (Fig 1B, compare pk8TR with m4TR lanes), indicating less episomal DNA in these cells. For m8TR DNA, episomes were detected in 10 of 17 lanes (Fig 1C), and in three experiments, 19 of 50 (38%) of G418 resistant cell lines had episomes. Although the m8TR episomal signal was weaker compared to that of the pk8TR cells (Fig 1C), there was substantially more m8TR episomal signal compared to that of m4TR (Fig 1B). Probe used in Fig 1B and 1C detected only vector backbone and therefore the efficiency of detection for all plasmids was the same. In general, mTR episomes migrated much more slowly than the ccc plasmid DNA (e.g. Fig 1B, lane 3 or Fig 1C, lane 2), and also more slowly than most pk8TR episomal DNA at 27 days of selection. The large, recombinant episomes were due to recombination events, and were primarily comprised of input plasmids arranged in tandem head to tail multimers, with expansion and contraction of tandem mTRs also occurring (S1 Text, S1 and S2 Figs). Therefore, kLANA can mediate episome persistence of mTR DNA and the efficiency of episome persistence is greater with higher mTR copy number.

**Table 1:** Episome maintenance of mTR or kTR DNA in mLANA or kLANA expressing cells.

Cell line	Transfected DNA	Episome positive (%)^1^
BJAB-kLANA^2^	pk8TR	19/19 (100%)
BJAB-kLANA	m2TR	1/31 (3%)
BJAB-kLANA	m4TR	12/37 (32%)
BJAB-kLANA^2^ BJAB-kLANA^2^	m8TR pRepCK	19/50 (38%) 0/16 (0%)
BJAB	m2TR	0/7 (0%)
BJAB	m4TR	0/9 (0%)
BJAB	m8TR	0/14 (0%)
A20-mLANAF(A)^3^	m4TR-P	5/20 (25%)
A20-mLANAF(B)^4^	m4TR-P	48/83 (58%)
A20-mLANAF(B)	pRepCK-P	0/21 (0%)
A20^3^	m4TR-P	0/34 (0%)
A20-mLANAF(A)	pk8TR-P	2/3 (67%)
A20-mLANAF(B)	pk8TR-P	27/29 (93%)
A20	pk8TR-P	0/10 (0%)

^1^ Fractions indicate number of cell lines containing episomes divided by the total number of cell lines assayed by Gardella analyses; percentages are in parenthesis.

^2^Data from three experiments

^3^Data from two experiments.

^4^Data from five experiments.

We investigated kLANA’s ability to maintain mTR episomes over a longer time period. Of four cell lines (Fig 1B, cell lines b, e, f, i) that initially had m4TR episomes after 27 days of G418 selection, three (Fig 1B, cell lines b, e, f) no longer contained episomes after ~2 months in continuous culture and the fourth m4TR cell line (Fig 1B, cell line i) had lost nearly all episomal DNA after 87 days of G418 selection (Fig 1D, lane 19, asterisk indicates faint band). In contrast, eight G418 resistant m8TR cell lines containing episomal DNA at 27 days continued to maintain m8TR episomal DNA after 87 days of selection (Fig 1D), while, as expected two cell lines which lacked episomes at 27 days, cell lines a and e, remained negative. Therefore, kLANA acts on m8TR DNA to mediate longterm episome persistence for at least ~3 months whereas m4TR episomal DNA was lost over time.

### kLANA redistributes and concentrates to dots along mitotic chromosomes in the presence of mTR episomes

kLANA was detected in BJAB cells expressing kLANA either in the presence or absence of episomes that had been under G418 selection for over three months. Both interphase and metaphase arrested cells were assessed (Fig 1E). As expected, in the absence of episomes, kLANA (green) distributed broadly throughout the nucleus (red) in interphase (overlay of red and green generates yellow) and over mitotic chromosomes (red). In contrast, kLANA (green) concentrated to dots both in interphase nuclei (red), and along mitotic chromosomes (red) in cells containing k8TR episomes. Previous work demonstrated that each kLANA dot colocalizes with an episome[7]. In cells with m8TR episomes (from Fig 1C and 1D, cell line d used as a representative example), kLANA concentrated to dots in interphase and along mitotic chromosomes (red), but some kLANA also broadly distributed throughout the nucleus in interphase and broadly along mitotic chromosomes. In addition, there were generally fewer kLANA dots in cells with m8TR compared with k8TR, consistent with fewer m8TR episomes per cell. Therefore, kLANA relocalized to dots in both k8TR and m8TR cells, although the relocalization was less pronounced in cells with m8TR episomes compared with k8TR episomes.

### mLANA acts *in trans* on kTR DNA to mediate episome persistence

Since kLANA mediated episome persistence of mTR DNA, we asked if mLANA can mediate episome persistence of k8TR DNA. mLANA acts on mTRs to mediate episome persistence when both are *in cis*[33], and we first performed experiments that demonstrated mLANA also acts *in trans* to mediate persistence (S3 Fig, S2 Text). To assess if mLANA mediates episome persistence of kTR DNA, pk8TR-P or pRepCK-P vector (encoding puroymcin resistance) was transfected into murine A20 cells or A20 cells expressing mLANAF. As expected, puromycin resistant outgrowth was low after transfection of vector pRepCK-P or pk8TR-P into A20 cells (S1 Table). In contrast, transfection of pk8TR-P into A20-mLANAF cells resulted in much higher outgrowth, consistent with episome maintenance.

**Fig 2:**
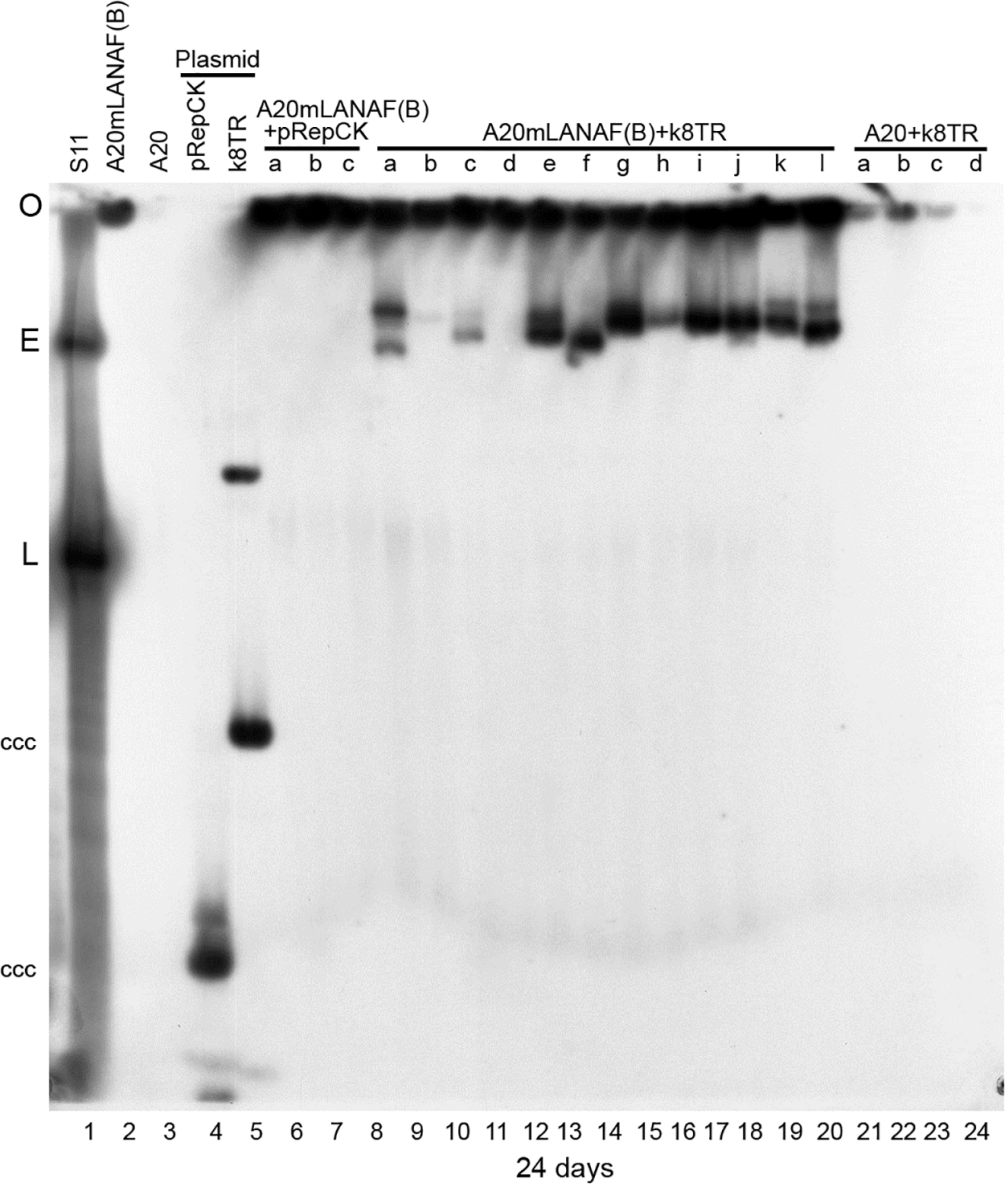
mLANA mediates kTR episome persistence. Gardella gel after transfection of A20 or A20/mLANA cells with pRepCK vector or k8TR DNA. Lanes contain 2-3x10^6^ cells. Gel was performed at 24 days of puromycin selection. Blot was probed with ^32^P-pk8TR DNA. O, gel origin; E, S11 episomes; L, S11 linear genomes due to lytic replication; ccc plasmid DNA is indicated.

Cells were assessed by Gardella gel for episomes. As expected, mLANA expressing cells transfected with vector control or A20 cells transfected with k8TR-P lacked episomes (Fig 2). In contrast, after transfection of pk8TR-P into A20-mLANAF(B) cells, 11 of 12 (Fig 2) cell lines had episomes and in a total of 4 experiments, 27 of 29 (93%) of puromycin resistant cells contained episomes (Table 1). Therefore, mLANA mediates episome persistence of k8TR DNA at relatively high efficiency. Consistent with these results, and with those that showed kLANA mediates episome persistence of mTR DNA (Fig 1), we found that kLANA and mLANA bind reciprocally to each other’s TR DNA recognition sequences (S3 Text, S4 Fig).

### Generation of kLANA-MHV68 chimeric viruses

**Fig 3:**
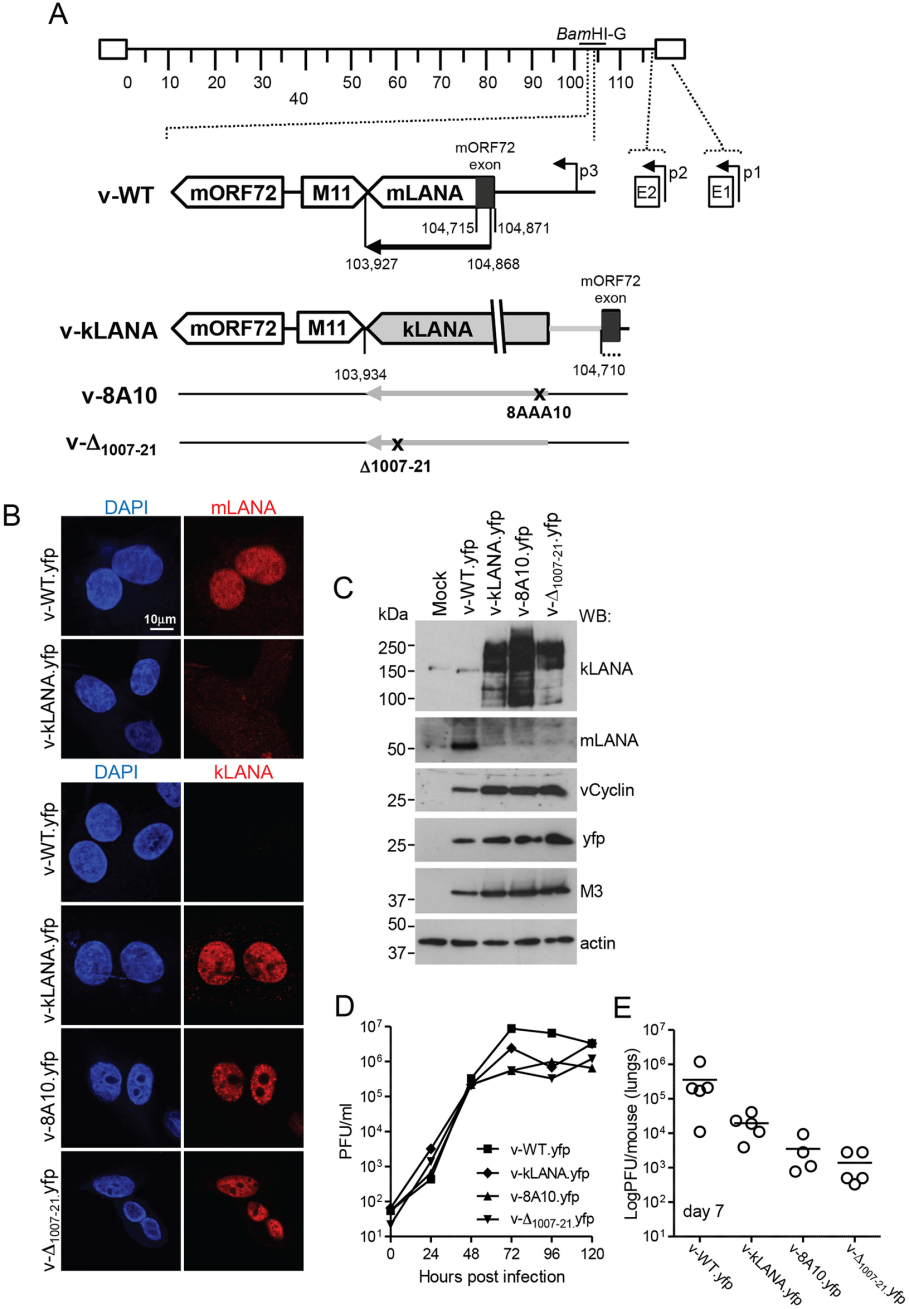
Generation and lytic growth of MHV68 chimeric viruses. (A) Schematic diagram. The kLANA cassette was inserted between the M11 stop codon and the mORF72 exon in place of MHV68 103,935–104,709, which includes most of the mLANA ORF. p1, p2, p3, are mLANA promoters[38,39]. The mORF72 noncoding exon (black) is located within the mLANA coding region. The E2 splice acceptor site (nt 104,871) and the mORF72 exon splice donor site (nt 104,715) were left intact to ensure expression of kLANA and mORF72. The mLANA start codon and three downstream ATGs were mutated to ATT to prevent initiation of translation (indicated by black dots). The BamHI-G fragment (genomic nt 101,653–106,902) is indicated. mLANA ORF, nt 104,868–103,927. (B) Confocal immunofluorescence detection of mLANA (top panels) or kLANA (lower panels) from yfp viruses. Magnification 630x. (C) Immunoblot of viral proteins. (D) Growth curves of virus in BHK-21 cells after infection with 0.01 PFU/cell. There was no significant difference between infection groups (p>0.05 using one-way non-parametric ANOVA Kruskal-Wallis). (E) Lung virus titers 7 days after infection with 10^4^ PFU of the indicated viruses. Circles represent titers of individual mice (n = 19). Bars indicate the mean. v-Δ1007-21.yfp had significantly lower titers than v-WT.yfp (**p<0.01, using one-way non-parametric ANOVA Kruskal-Wallis followed by Dunn´s multiple comparison test). There were no other statistically significant differences between groups.

Since kLANA supported episome maintenance of mTR DNA, we asked if kLANA can functionally replace mLANA in MHV68 to support infection in vivo. The kLANA ORF and 5´ UTR, but not the kLANA promoter[37], were inserted into MHV68 at the mLANA locus. The mORF72 (noncoding) exon, which overlaps with N-terminal mLANA, was retained to preserve splicing events important for expression of mORF72 and LANA. Thus, kLANA expression is driven by native mLANA promoters (Fig 3A)[38,39] in the absence of mLANA. The resulting chimeric virus was termed v-kLANA. We also engineered viruses with mutations in kLANA that abolish nucleosome binding (termed v-8A10, where LANA residues _8_LRS_10_ were mutated to _8_AAA_10_)[40] or LANA DNA binding (termed v-Δ_1007–21_, where residues 1007 to 1021 were deleted) [41] (Fig 3A). These mutations abolish kLANA episome persistence[40,41]. All kLANA-MHV68 recombinants were generated in backgrounds of wild type MHV68 or yellow fluorescent protein (yfp) MHV68[42].

### Lytic replication of kLANA MHV68 viruses

mLANA promoters are predicted to drive transcription of transgenic kLANA during lytic replication in vitro, as they do for mLANA and mORF72[38,43]. We assessed LANA expression after infecting BHK-21 cells with MHV68 (v-WT), v-kLANA, v8A10, or v-Δ_1007–21_. As expected, v-WT expressed mLANA, which distributed broadly throughout the nucleus (Fig 3B and S5A Fig, top panels)[43]. kLANA or kLANA mutants distributed similarly to mLANA (Fig 3B and S5A Fig, bottom panels) after infection with the chimeric viruses. Immunoblots confirmed mLANA or kLANA expression after infection with WT or chimeric viruses (Fig 3C, S5B Fig). The multiple kLANA bands are due to alternative initiation of translation and an alternative poly adenylation signal[44,45]. vCyclin (ORF72) and M3 (a chemokine binding protein expressed in lytic infection) levels were similar for v-WT and chimeric viruses (Fig 3C, S5B Fig), indicating preserved expression and comparable infection levels.

Growth kinetics of v-kLANA, v8A10, or v-Δ_1007–21_ in BHK-21 cells infected at MOI of 0.01 was similar to that of v-WT virus (Fig 3D and S5C Fig). To assess lytic replication in vivo, we inoculated by intranasal (i.n.) route C57 BL/6 mice with 10^4^ PFU of v-WT or v-kLANA virus and determined lung titers. While titers of all viruses were similar at day 3 (S5D Fig), at day 7 after infection titers were slightly lower for v-kLANA viruses, particularly for the kLANA mutants, compared to v-WT (Fig 3E and S5D Fig). At day 14 no virus was detected in the lungs (S5D Fig). The reduction of titers at day 7 indicates that kLANA cannot fully replace mLANA during lytic replication in vivo, and disruption of kLANA N-terminal chromosome binding or C-terminal DNA binding affected this phase of infection.

### kLANA rescues MHV68 in vivo latent infection in the absence of mLANA

**Fig 4:**
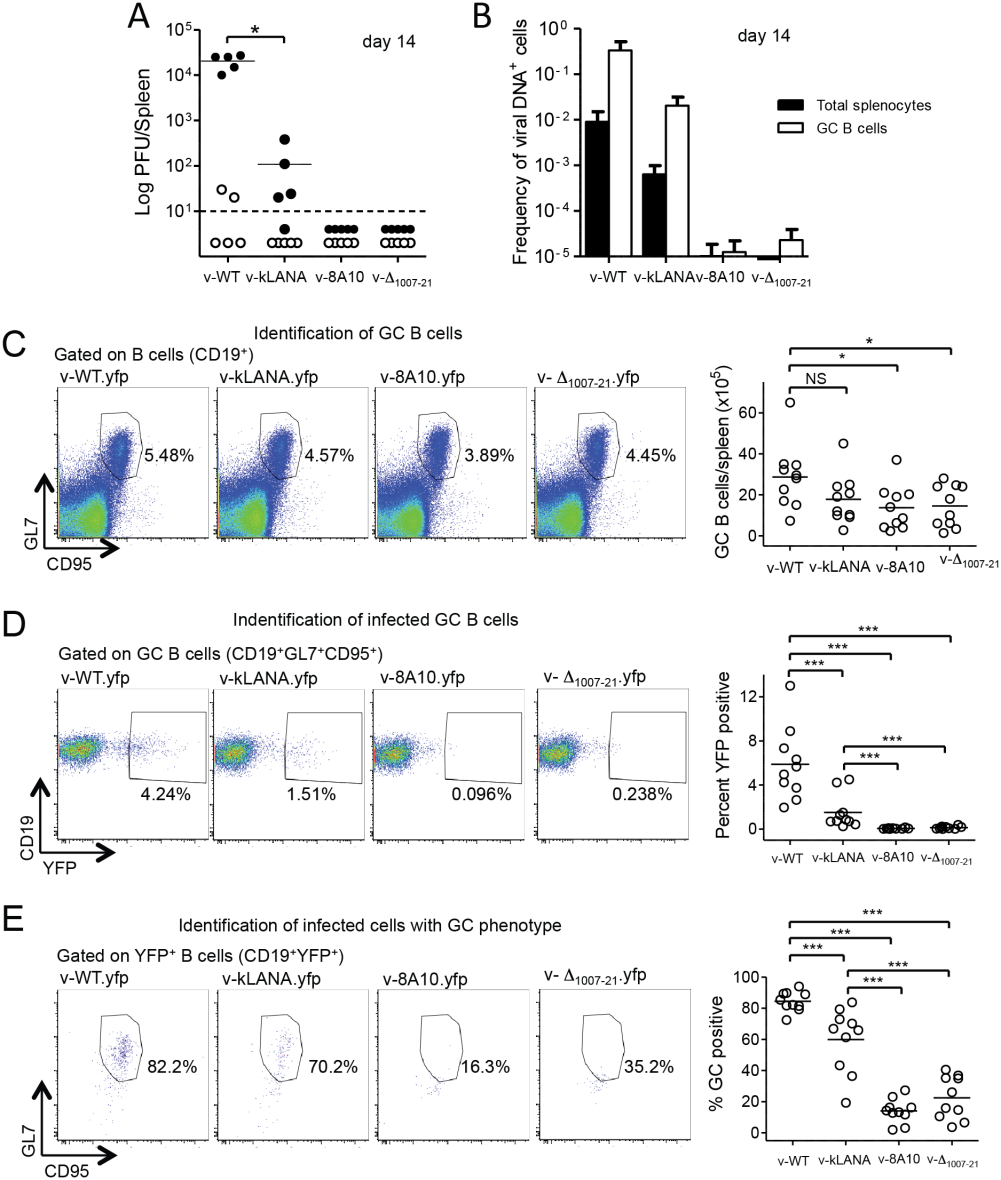
v-kLANA latent infection. Viral latency in spleens of C57 BL/6 mice 14 days after i.n. infection with 10^4^ PFU of the indicated viruses. (A) Latent titers determined by co-culture reactivation assay (closed circles) and titers of pre-formed infectious virus by plaque assay (open circles). Circles are titers of individual mice. Bars indicate mean and dashed line shows the limit of assay detection. v-kLANA titers were significantly lower than v-WT (Mann-Whitney test). *p<0.05. (B) Quantification of viral DNA-positive cells in total splenocytes and in sorted GC B cells (CD19^+^CD95^+^GL7^+^). Data are from pools of five spleens per group. Bars are frequency of viral DNA-positive cells. Error bars indicate 95% confidence intervals. (C-E) Flow cytometry analyses. Representative FACS plots from individual mice are shown in left panels. Quantification graphs in which each point represents an individual mouse are shown at the right. Bars are mean values. Data were combined from 2 independent experiments with 5 mice in each group. (C) Total number of GC B cells (CD19^+^CD95^+^GL7^+^). NS, not significant; *p<0.05 using the Mann-Whitney test. (D) Percentage of GC B cells that were YFP positive. (E) Percentage of YFP positive cells that were GC B cells. ***p<0.001 in (D) and (E) using the Mann-Whitney test.

To assess if kLANA can support MHV68 latent infection in vivo we assessed viral latency in spleens of mice infected with v-WT or kLANA chimeric virus. To identify latently infected splenocytes at day 14 after infection (the peak of latent infection), cells were incubated with BHK cells (permissive for lytic infection) to detect virus produced after lytic reactivation. As expected, splenocytes from v-WT infected mice produced virions. Splenocytes from v-kLANA infected mice also produced virus, although titers were 2 to 2 ½-log lower than those of v-WT infected animals (Fig 4A). In striking contrast, no detectable reactivating virus was observed in the v-8A10 or v-Δ_1007–21_ infection groups (Fig 4A).

We also investigated the frequency of infected cells by limiting dilution PCR, in total splenocytes or in germinal centre (GC) B cells, since this approach directly indicates numbers of latently infected cells in the absence of a requirement for lytic reactivation. The frequency of latently infected splenocytes or GC B cells for v-kLANA was ~1 log lower compared to that of v-WT. In direct contrast, latently infected cells were at much lower levels for v-8A10 or v-Δ_1007–21_ (Fig 4B).

We assessed the possibility that the reduction in v-kLANA latent infection at day 14 could be due to differing kinetics of infection of v-kLANA compared to v-WT by examining levels of latency at days 11 and 21. Results demonstrated that v-kLANA latent infection was similarly reduced compared to v-WT at days 11 and 21, indicating a consistent deficiency over time rather than differing infection kinetics (S6 Fig). We also asked if the lower level of latently infected splenocytes might be due to v-kLANA’s reduced lytic replication in the lungs. However, after intraperitoneal infection, which bypasses the lungs to provide virus access to the spleen, latent infection remained lower for v-kLANA compared to v-WT (S6A and S6B Fig).

We assessed latent infection and GC B cell populations in the spleen by flow cytometry 14 days after infection with WT or kLANA yfp viruses. The percentage of B cells that were GC B cells varied between ~3–6% among infection groups (Fig 4C). Total number of GC B cells was slightly higher in mice infected with WT when compared to kLANA, and significantly lower numbers were observed for the kLANA mutants compared to v-WT (Fig 4C, right panel). This result is not unexpected, in light of results in Fig 4A and 4B since the magnitude of GC B cell amplification after MHV68 infection correlates with latent virus load[46]. The frequency of GC B cells that were infected was determined from YFP expression. Mean percent of YFP^+^ GC B cells was 5.8% for v-WT.yfp and 1.4% for v-kLANA.yfp infected mice (Fig 4D, right panel). Mice infected with the kLANA mutant yfp viruses had over 10-fold lower percent of YFP^+^ GC B cells compared with v-kLANA.yfp (Fig 4D). About 80% of v-WT.yfp infected cells infected had a GC phenotype. Similarly, ~60% of v-kLANA.yfp infected cells had a GC phenotype (Fig 4E, right panel). In contrast, of the very few infected YFP+ B cells in the v-8A10.yfp and v-Δ_1007-21_.yfp groups, only small percentages, 14% and 22%, respectively, were GC B cells (Fig 4E, right panel). Thus, kLANA largely rescued MHV68 in vivo latency in the absence of mLANA, whereas kLANA containing mutations that abolish episome persistence did not.

### kLANA chimeric virus persists at WT levels in latently infected cells

**Fig 5:**
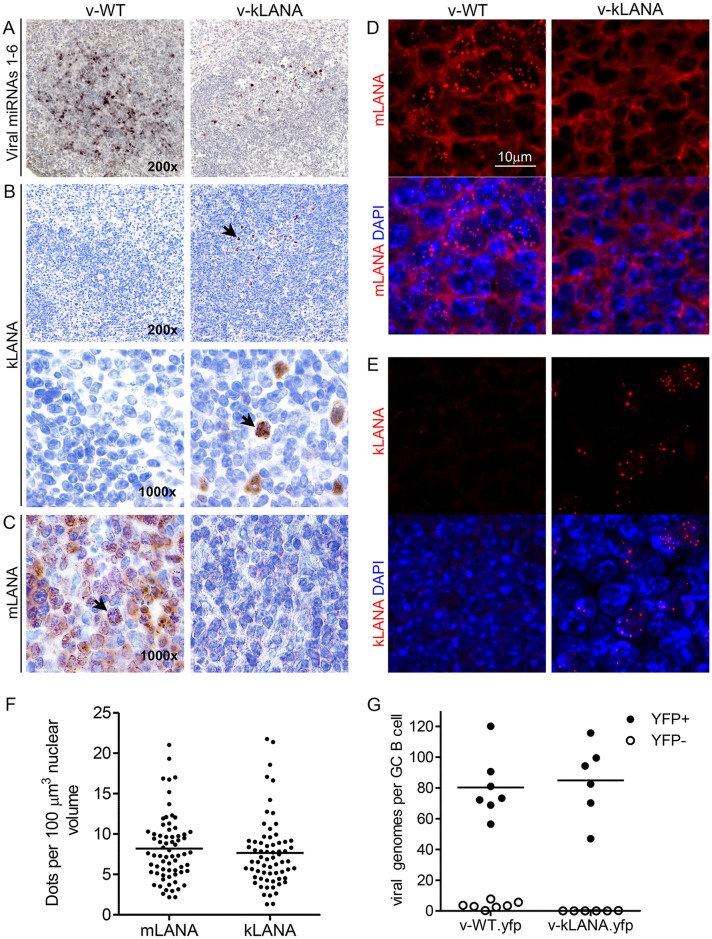
mLANA and kLANA expression in vivo. Spleen sections of mice infected with 10^4^ PFU of v-WT or v-kLANA for 14 days. (A) In situ hybridization (brown) with probes for viral miRNAs 1–6. Sections were counter stained with Mayer´s Haemalum. (B, C) Detection of kLANA (B) and mLANA (C) by immunohistochemistry in sections adjacent to those shown in panel A. Arrows in panel B indicate the same kLANA positive cell. Arrow in panel C indicates a mLANA positive cell. Sections were counterstained with haematoxylin. No kLANA signal was detected in sections stained only with secondary antibody. (D, E) mLANA and kLANA nuclear dots detected by indirect immunofluorescence. Images are maximum intensity projections of Z-stacks acquired over the thickness of the spleen sections. No dots were observed in unstained sections or with secondary antibody alone. Magnification 630x. (F) Quantification of mLANA (n = 69 nuclei from 3 mice) or kLANA (n = 67 nuclei from 3 mice) dots per 100 μm^3^ nuclear volume. Bars indicate means. The number of dots per volume was not significantly different between v-WT and v-kLANA mice (Mann-Whitney test, p>0.05). (G) Viral genomes in FACS sorted YFP^+^ and YFP^-^ GC B cells from spleens of v-WT.yfp (n = 7) and v-kLANA.yfp (n = 6) infected mice. Circles represent individual mice. Bars indicate means. There was no significant difference between the two infection groups (Mann-Whitney test, p>0.05).

To further investigate v-kLANA latency we assessed kLANA expression in vivo. We first confirmed latent v-kLANA infection in spleen sections by detection of MHV68 miRNAs 1–6, which are expressed in latently infected cells[30]. As expected, signal was detected in v-kLANA infected mice, although in fewer cells compared to v-WT (Fig 5A). LANA concentrates to dots at sites of episomal DNA in nuclei of latently infected cells[7]. kLANA was detected by immunohistochemistry, sometimes as nuclear dots, in adjacent sections in B cell follicles of v-kLANA mice (Fig 5B, arrow). mLANA distributed similarly in nuclear dots in v-WT sections (Fig 5C). We also detected LANA dots by immunofluorescence in v-WT (Fig 5D) or v-kLANA (Fig 5E) infected B cell follicles.

We quantified the number of nuclear dots since LANA concentrates to dots at viral episomes, and each dot therefore indicates a viral genome. Since entire nuclei were rarely present in sections, we counted the number of dots per nuclear volume across confocal z-stacks. The concentration of mLANA or kLANA dots was similar, with means of 8.2 (range 2.2–21.0) and 7.7 (range 1.3 to 21.8) dots per 100μm^3^, respectively (Fig 5F). Considering a 4:1 nucleus-cytoplasm volume and diameters of 7 to 10 μm for GC B cells[47], we estimate a nuclear volume of 144 to 419 μm^3^ for the infected cells. Based on these volumes, we predict a mean of 12 to 34 mLANA or kLANA dots, hence viral episomes, per nucleus in latently infected cells within B cell follicles. We also quantified viral genomes in FACS sorted GC B cells from infected mice by qPCR. In YFP^+^ GC B cells the mean number of copies per cell was 80.6 (range 56.5 to 120.1) and 84.9 (range 47 to 115.7) for v-WT.yfp and v-kLANA.yfp, respectively (Fig 5G). The greater number of epsiomes determined by PCR could relate to an underestimation of nuclear volume when calculating LANA dots per nucleus, or possibly to adjacent genomes being observed as single dots. Together these results indicate that kLANA chimeric virus persists at WT copy number in nuclei of latently infected splenocytes.

## Discussion

This work demonstrates that kLANA and mLANA act reciprocally to mediate episome persistence of TR DNA. This functional conservation provided the rationale to assess a chimeric MHV68, with kLANA substituting for mLANA. kLANA rescued mLANA deficiency, with chimeric virus establishing latent infection in vivo. These findings were not necessarily expected. Previous work showed that the rhesus gamma-1 herpesvirus EBNA1 episome maintenance protein acts on the human Epstein-Barr virus (EBV) oriP element in vitro to support episome maintenance[48]. However, the gamma-1 herpesviruses are more closely related compared to the gamma-2 herpesviruses. Gamma-1 herpesviruses infect only humans (EBV) and non human primates[49] and the rhesus gamma-1 herpesvirus is estimated to have diverged from EBV only ~5 million years ago[50]. In contrast, gamma-2 herpesviruses infect humans (KSHV), non human primates, and many non primate species. Correspondingly, MHV68 is estimated to have diverged from KSHV ~60 million years ago[49,50]. Consistent with this degree of evolutionary divergence, mLANA and kLANA differ substantially in sequence (Fig 1A) and also complex differently with DNA[51]; kTR (0.8kb) and mTR (1.2 kb) also differ in size and lack sequence homology, although both are GC rich (84.5% and 77.6% GC for kTR and mTR, respectively)[32]. In agreement with the findings here, functional conservation was observed for kLANA’s ability to repress transcription from the mTR, similar to mLANA[52].

The modest attenuation phenotype of v-kLANA in latent infection compared to v-WT could be due to a deficiency in lytic reactivation or to LANA related growth effects. v-kLANA exhibited a 2 log deficiency compared to v-WT in a reactivation based assay (Fig 4A). However, the frequency of v-kLANA latently infected GC B cells was only ~1 log lower (Fig 4B), although it is important to note that the number of GC B cells was modestly decreased in v-kLANA compared to v-WT infected mice (Fig 4C). It remains possible that lytic reactivation may be important to expand the number of infected cells early in latency establishment. However, it is also possible that kLANA fails to fully promote efficient proliferation of infected GC B cells through functions such as mLANA’s regulation of protein levels of Myc and NF-kb through ubiquitination[53,54].

Despite a decrease in the number of infected GC B cells, kLANA fully recapitulated mLANA’s ability to maintain a WT genome copy number in cells. Episome copy number was similar for v-WT.yfp and v-kLANA.yfp whether determined by the number of mLANA or kLANA dots per nucleus (each LANA dot corresponds to an episome) (Fig 5F) or by PCR of virus DNA from infected cells (Fig 5G). Although these approaches resulted in modestly different absolute genome copy number estimations, both indicate that kLANA and mLANA infected GC B cells harbor equivalent number of episomes per cell.

The small deficit in lung replication of kLANA chimeric virus suggests that kLANA does not fully replace mLANA in this phase of infection. In previous work, mLANA-null viruses or recombinants harboring C-terminal mLANA mutations that abolish DNA binding, had small lytic growth defects in cultured murine fibroblasts[43,52] and an attenuation in acute lytic lung replication[34,55,56]. Thus, mLANA and in particular the C-terminal DNA binding domain, was deemed required for efficient lytic replication. The lung attenuation phenotype of v-Δ_1007–21_, which encodes a DNA binding deficient kLANA, is reminiscent of these findings[55].

The finding that kLANA mutations 8A10 or Δ_1007–21_ abolish MHV68’s ability to efficiently establish latency (Fig 4), demonstrates episome persistence is necessary for latency establishment. LANA 8A10 or Δ_1007–21_ each abolish LANA’s ability to replicate DNA and segregate episomes to progeny nuclei by eliminating chromosome association or DNA binding, respectively. These results indicate that the critical function of these kLANA residues is conserved in this chimeric infection model.

This work suggests differences in episome maintenance efficiency between kLANA and mLANA. Although both kLANA and mLANA act on TR DNA to mediate episome persistence, kLANA appeared to act more efficiently on cognate DNA than did mLANA. kLANA maintained episomes in 100% (Table 1) of G418 resistant cell lines after transfection of k8TR DNA, while mLANA maintained episomes in only 25%-58% of puromycin resistant cell lines after transfection of m4TR-P DNA. The use of four mTR copies versus eight kTR copies in these experiments is unlikely to account for the substantially lower mLANA efficiency. In fact, in prior work, kLANA consistently maintained episomes in all G418 resistant cell lines after transfection of plasmids containing two, three, or eight kTR copies ([8,40,57]).

It is possible that mLANA’s diminished efficiency relates to inherent differences in the TR elements rather than in differences between mLANA and kLANA. As expected, kLANA mediated episome persistence more efficiently for kTR than for mTR DNA (Table 1). However, mLANA also mediated episome persistence more efficiently for kTR compared to mTR. mLANA mediated persistence of k8TR DNA in 67–93% of cell lines, while persistence of m4TR DNA occurred in only 25–58% of cell lines. Although this difference may be related to the additional 4 TR elements in k8TR versus m4TR, it is possible that KSHV TR elements have evolved to enable a higher level of episome maintenance efficiency.

kLANA and mLANA demonstrate reciprocal binding to each other’s recognition sequences, although binding of kLANA to mLBS is substantially weaker than binding to kLBS (S4 Fig). Although kLANA and mLANA DNA binding sites share substantial homology, they are not identical (S4A Fig), and mLBS differs from the high affinity kLBS1 at several nucleotides important for LANA binding [55,58]. Consistent with these findings, the kLANA DBD binds mLBS1-2 with a *K*_*D*_ of 116.0 nM compared to 13.7 nM for kLBS1-2 [51]. kLANA’s diminished binding to mLBS may also relate to its inherently different thermodynamic binding mode compared to mLANA.

Despite the less efficient binding of kLANA to mLBS, kLANA enabled episome persistence in spleen follicles with virus persisting at WT levels in infected cells, as evidenced by the number of viral genomes per infected cell. kLANA and mLANA DNA binding domains share significant structural homology and interact to form oligomerized dimers [55,59–61]. It is likely that the presence of multiple TR elements (each of which contains LBS1-2) in MHV68 results in enhanced cooperative binding and/or other oligomerization events that increase binding efficiency which may allow kLANA to efficiently mediate episome maintenance of mTR DNA and virus persistence. For instance, despite mutations that engendered severe mLANA DNA binding deficiency to mLBS1-2, mLANA efficiently mediated episome persistence in the setting of a full complement of mTRs in MHV68 [62]. Further, the finding of large, recombinant 4TR and 8TR episomes here (Figs 1 and 2; S1 and S2 Figs), and as previously observed[7,63,64] likely reflects the need for ~40 TR elements, similar to that of KSHV, for optimal functional efficiency.

Here, we show that despite more than 60 Ma of evolutionary divergence, mLANA and kLANA exhibit reciprocal episome maintenance function, providing the basis for in vivo analysis of KSHV LANA. This chimeric virus should allow for future investigation of kLANA in a well-established small animal model of MHV68 latent infection. The in vivo model also provides a means to assess kLANA targeting through strategies such as small molecule inhibition. Last, this approach potentially can be applied to other viruses which lack small animal models, but for which model virus systems exist.

## Materials and methods

### Cell lines

A20 murine B lymphoma cells[65] (ATCC) were cultured with RPMI supplemented with 10% Fetalplex (Gemini) or bovine growth serum (BGS) (Hyclone), beta mercaptoethanol, sodium pyruvate, HEPES, Glutamax (Invitrogen), and 15μg/ml gentamicin. The latently infected cell lines, S11 (MHV68 infected)[66] and BCBL-1 (KSHV infected) (NIH AIDS Reagent Program) were maintained in RPMI with 20% BGS. S11 cells were also supplemented with beta mercaptoethanol. BJAB cells were grown in RPMI medium containing 10% BGS (Hyclone) or Fetalplex (Gemini) and 15μg/ml gentamicin. BJAB cells stably expressing KSHV LANA containing an N-terminal FLAG epitope tag (BJAB-kLANA)[7] were grown in RPMI containing 10% Fetalplex or BGS, and medium was supplemented with hygromycin B (200 units/ml; Calbiochem). 293T (ATCC) cells were grown in DMEM medium containing 10% BGS. NIH-3T3-CRE cells[67] were grown in Dulbecco’s modified Eagle’s medium (DMEM) supplemented with 10% heat inactivated fetal bovine serum, 2mM glutamine, and 100U/ml penicillin and streptamicin. Baby hamster kidney fibroblasts (BHK-21, clone 13 CCL-10) (ATCC) were cultured in Glasgow´s modified Eagle´s medium (GMEM) supplemented as for NIH-3T3-CRE cells with the addition of 10% tryptose phosphate buffer. Cells were confirmed to be mycoplasma free.

### Plasmids

mLANAF[33] contains mLANA with a 3x C-terminal FLAG tag driven by its native promoter. mLANA with N-terminal Myc and C terminal 3×FLAG epitope tags was generated by digesting pCMV-myc-mLANA-C3F with SacI and XhoI, and the resulting mLANA fragment ligated into pBluescript+II vector digested with SacI and XhoI. m2TR, m4TR and m8TR[33] contain two, four or eight mTR copies of the MHV68 terminal repeat elements. To generate m4TR-P, the neomycin resistance gene was removed from m4TR by digestion with BglII and HpaI and the BglII site blunted. The puromycin resistance gene was removed from pBabe puro[68] by digestion with ClaI and SnaBI, the ClaI site blunted, and the fragment inserted into the HpaI and BglII digested m4TR, generating m4TR-P. To generate pRepCK-P, the mTRs were removed from pm4TR-P by Not I digestion and the NotI site religated. To generate pk8TR-P, p8TR (pk8TR)[40] was digested with MluI and PsiI to release the neomycin resistance gene and then ligated with the MluI/PsiI DNA fragment containing the puromycin resistance gene from pm4TR-P.

### Western blot and antibodies

Extracts derived from 0.25x10^6^ puromycin resistant cells were loaded per lane for FLAG antibody blots and 0.5x10^6^ cells per lane were loaded for the blot probed with anti-mLANA monoclonal antibody 6A3 in S3 Fig. Murine monoclonal antibody 6A3[55], which was raised against mLANA amino acids 140–314, was generated at the Monoclonal Antibody Core Facility, European Molecular Biology Laboratory. Proteins were resolved by 8% SDS-PAGE, transferred to nitrocellulose, and detected with anti-FLAG antibody conjugated to HRP (Sigma) used at a 1:750 dilution, mouse anti-tubulin monoclonal antibody B-5-1-2 (Sigma) used at a 1:1000 dilution, or 6A3 hybridoma supernatant used at a 1:2 dilution. Secondary anti mouse HRP conjugated antibody followed by chemiluminescence was used to detect the anti-tubulin and 6A3 antibodies. Anti-kLANA rat monoclonal antibody (LN53, ABI Sciences) was used at 1:1000, anti-vCyclin (mORF72)[69] (a gift from Samuel Speck) was used at 1:500, rabbit anti-M3 polyclonal[70] was used at 1:3000, mouse anti-eGFP (Clontech) was used at 1:1000 and rabbit anti-actin (Sigma) was used at 1:1000. Horse radish peroxidase (HRP) conjugated secondary antibodies were from GE Healthcare and Jackson Immunoresearch.

### Generation of A20 cells with stable expression of mLANA

Ten million A20 cells in log phase were transfected with 35 μg of mLANAF or 35 μg of pRepCK vector in 400 μl RPMI containing 10% serum, but without antibiotics, by electroporation using the BTX Electroporation System Electrosquare Porator T820 by pulsing the cells at 225 volts for 65 milliseconds once. Three days post transfection, cells were seeded into microtiter plates at 5000, 1000, or 100 cells per well and placed under G418 (400 μg/ml) (Gemini) selection. G418 resistant clones were expanded and those transfected with mLANAF were screened for mLANAF expression by immunoblot with anti-FLAG antibody.

### Episome maintenance assays

G418 resistant A20 cells or G418 resistant A20 cells stably expressing mLANAF were transfected as above with 35 μg of m4TR-P, pk8TR-P, or pRepCK-P and cultured in RPMI supplemented with 400 μg/ml G418 (Gemini) for 3 days. Cells were then seeded into 96 well plates at 10, 100, or 1000 cells per well and puromycin 2.5μg/ml (Invitrogen) was included in the medium in addition to G418. Cell lines resistant to both puromycin and G418 were expanded.

BJAB-kLANA cells were grown in log phase for three consecutive days and then ten million cells were transfected with 35 μg of m2TR, m4TR, m8TR, pk8TR, pRepCK, or plasmid DNA rescued from episome containing cells, in 400 μl of RPMI medium with 10% serum at 200 V and 960 μF in a 0.4-cm-gap cuvette with a Bio-Rad electroporator. Three days post transfection, cells were seeded in micro titer plates at 1, 10, 100, or 1000 cells per well in medium containing G418 (600 μg/ml; Gibco or Gemini) and later expanded to 6 well plates.

Gardella gel analysis[71] was performed on A20 or BJAB cell lines. Cells were lysed *in situ* in gel-loading wells embedded with DNAse free protease (Sigma #P6911) and sodium dodecyl sulfate, and electrophoresis in Tris-borate-EDTA performed. DNA was then transferred to a nylon membrane and detected by autoradiography using ^32^P-labeled probe.

### Fluorescence microscopy of suspension cells

G418 resistant BJAB-kLANA cells transfected with m8TR, k8TR or pRepCK, were metaphase arrested by incubation for 16 hours with 1 μg/mL of colcemid (Calbiochem). 0.2 x10^6^ cells were resuspended in 1 mL hypotonic buffer (1% NaCitrate, 1mM MgCl_2_, 1mM CaCl_2_) for 5 minutes. 300 μL cells were spread onto a polylysine slide by cytopsin (Thermoshandon), fixed in 4% formaldehyde, permeabilized with 0.5% triton X-100 and blocked in 20% goat serum. kLANA was detected by incubating with anti-kLANA monoclonal antibody IA-2-12 (gift of Mary Ballestas)[72] diluted 1:1000 in 20% goat serum for two hours, followed by incubation with secondary anti-mouse antibody conjugated to Alexa Fluor 488 (Molecular Probes) diluted 1:1000 in 20% goat serum. DNA was detected using propidium iodide at 1 μg/mL (Invitrogen). Slides were dried in ethanol 70% followed by incubations in 90% and 100% ethanol before coverslips were applied with Aqua-Poly mount (Polysciences). Microscopy was performed with a Zeiss Axioskop, PCM2000 hardware, and C-imaging software (Compix, Inc.).

### Recombinant viruses

DNA encoding the kLANA gene and 5´UTR, flanked by MuHV4 genomic sequence, was cloned into pSP72 (Promega). The 5´ end of mORF73 and upper flanking region (coordinates 104710–105092, GenBank accession U97553) were PCR amplified from the MuHV-4 genome with primers Imm_TR1 (AAAGAATTC**A**ATCACCTTGGCATCC) and Imm_TR2 (AATGCCTGAAGATCTTCCAG). Primer Imm_TR1 introduces an EcoRI site (underlined) four bases downstream of the mORF72 splice donor site (double underlined) and a C104710A mutation (bold) to alter ATG (coordinate 104712) to ATT. Primer Imm_TR2 contains the BglII genomic site (coordinate 105087). The PCR fragment was cloned into the pSP72 using BglII /EcoRI sites to create pSP72_PCR1. The mORF73 ATG (coordinate 104869) and two downstream ATG sequences (coordinates 104779 and 104714) were altered to ATT in pSP72_PCR1 using QuikChange Multi Site-Directed Mutagenesis (Stratagene) and primers Imm_TR5_C104721A (GAATTCAATCACCTTGG**A**ATCCCGGTGGTGG), Imm_TR6_C104777A (GCGTCTTTTAGGAGG**A**ATGGCTGCTGGTTTG) and Imm_TR7_C104867A (CGGTGGGGATGTGGG**A**ATTATCTGAAAGAG) (mutated nucleotides in bold.) The resulting plasmid was termed pSP72_PCR1_2. The region of KSHV encoding the N-terminal region of kLANA and 5´UTR (coordinates 126473–127886, GenBank accession U75698) was PCR amplified from L54 phage[73] DNA with primers Imm_TR8 (ATCACCCCAGGATCCCTCAGAC), and Imm_TR9 (AAAGAATTCATTTGGAGGCAGCTGCG). Imm_TR8 contains genomic BamHI site (underlined, coordinate 126473) and Imm_TR9 introduces an EcoRI site (underlined) before the kLANA 5´UTR. The PCR product was ligated into the BamHI/EcoRI sites of pSP72_PCR2 to generate pSP72_PCR1_3. The DNA encoding the remainder of kLANA (corresponding to genomic coordinates 123808–126473) was amplified by PCR from pEGFP kLANA[40] with primers Imm_TR10 (AAAAAGCTT**TTA**TGTCATTTCCTGTGG) and Imm_TR11 (CTGAGGGATCCTGGGGTGATG). IMM_TR10 introduces a HindIII site (underlined) downstream of the kLANA stop codon (bold). Imm_TR11 contains a BamHI genomic site (underlined, coordinate 126473). The PCR product was ligated into pSP72_PCR1_3 using HindIII and BamHI sites to create pSP72_PCR1_4. The lower flanking region of mORF73 (coordinates 102728–103934) was obtained by PCR from MuHV-4 DNA with primers Imm_TR3 (AAACTCGAGCAGATGAGATCTGTACTC) and Imm_TR4 (AAAAAGCTTTCAGACATAAATCACATTC). Imm_TR3 introduces a XhoI site (underlined) 6 nucleotides from the genomic BglII site (coordinate 102728, double underlined) and IMM_TR4 introduces a HindIII site (underlined) after the M11 stop codon (bold). The PCR product was cloned into XhoI/HindIII sites of pSP72_PCR1_4 to generate pSP72_PCR1_5. Alanine substitutions 8LRS10 to 8AAA10 in kLANA were introduced by PCR into pSP72_PCR1_3 using primer kLANA_8AAA10 (CCGGGAATGCGC**GCCGCAG**CGGGACGGAGCACCGG) (mutated nucleotides in bold.) The mutated region was subcloned from pSP72_PCR1_3_8A into BamHI/SacI sites of pSP72_PCR1_5 to create pSP72_PCR1_5_8AAA10. DNA encoding kLANA Δ1007–21 was subcloned from pSG5 kLANA delta 1007–21[41] into pSP72_kLANA with BamHI and StuI to generate pSP72_PCR1_5_Δ1007–21. Sequence of PCR-derived regions was confirmed by DNA sequencing. The KSHV sequences flanked by MUHV4 genomic sequences from pSP72_PCR1_5, pSP72_8A and pSP72_Δ1007–21 were subcloned into the BamHI-G MuHV-4 genomic fragment cloned in the pST76K-SR shuttle plasmid, using BglII sites. Each recombinant BamHI-shuttle plasmid was transformed into E. coli harboring the WT MuHV-4 BAC (pHA3)[74] or yellow fluorescent protein (YFP)-WT MuHV-4 BAC[42]. Following a multi-step selection procedure[74], recombinant BAC genomes were identified by PCR and HindIII, BamHI and EcoRI digestion profiles. Viruses were reconstituted by transfection of BAC DNA into BHK-21 cells using X-tremeGENE HP (Roche Applied Science). The IoxP-flanked BAC cassette was removed by viral passage through NIH-3T3-CRE cells.

### Ethics statement

Animal studies were performed in accordance with the Portuguese official Veterinary Directorate (Portaria 1005/92), European Guideline 86/609/EEC, and Federation of European Laboratory Animal Science Associations guidelines on laboratory animal welfare. Animal experiments were approved by the Portuguese official veterinary department for welfare licensing (protocol AEC_2010_017_PS_Rdt_General), and the IMM Animal Ethics Committee.

### Viral stocks and in vivo assays

Virus stocks were prepared by infecting BHK-21 cells (0.001 PFU/cell). Viruses were harvested from cleared supernantants by centrifugation at 15000g for 2h at 4 C. Infectious virus titers were determined by plaque assay in BHK-21 cells. For experiments involving mice, sample size was based on our and other previous studies that produce biologically valid data. Specifically, sample size ranged from 3 mice per group to a maximum of 5 depending on the nature of the experiment. No specific randomization or blinding was applied. For in vivo infections, 6 to 8-week old C57BL/6 J female mice (Charles River Laboratories) were inoculated intranasally under isofluorane anaesthesia with 10^4^ of PFU in 20 μl PBS. At the indicated times after infection, mice were sacrificed and lungs or spleens harvested. Lungs were homogenized, freeze-thawed and titers determined by plaque assay. Single cell suspensions were prepared from spleens and reactivating virus was quantified by co-culture with BHK-21 cells. Plaque assay was performed in equivalent freezed-thawed samples to determine pre-formed infectious virus. Plates were incubated for 4 days (plaque assay) or 5 day (co-culture assay) then fixed with 4% formaldehyde and stained with 0.1% toluidine blue. Plaques were counted with a plate microscope.

### Flow cytometry

Single cell suspensions were prepared from spleens. After lysis of red blood cells in hypotonic NH_4_Cl solution, the number of viable cells was determined by trypan blue exclusion. Fc receptors were blocked with anti-CD16/32 (2.4G2) (BD Pharmigen) diluted in FACS buffer (PBS containing 2% fetal bovine serum) for 15 minutes on ice. Cells were incubated for 25 minutes on ice with the following antibodies diluted in FACS buffer: APC-H7-conjugated anti-CD19 (1D3) (BD Pharmingen) used at 1:400, PE-conjugated anti-CD95 (Jo2) (BD Pharmingen) used at1:800 and eF660-conjugated anti-GL7 (GL7) (eBioSciences Inc) used at 1:200. YFP expression and cell surface markers and were detected on a LSR Fortessa (BD BioSciences) using DIVA software and data were analysed with FlowJo 9.3.2 (Tree Star). Cells were gated on live cells and singlets based on FSC/SSC and FSC-A parameters, respectively. Total number of GC cells was determined based on FACS data and cell count of splenocytes.

### Frequencies of viral DNA positive cells

The frequency of viral DNA positive cells was determined in total splenocytes or GC B cells by limiting dilution combined with real time PCR. Splenocytes were pooled from 5 mice. GC B cells (CD19^+^GL7^+^CD95^+^) were purified from pools of 5 spleens using a BD FACSAria Fow Cytometer (BD BioSciences). The purity of sorted cells was≥ 95%. Cells were serially two-fold diluted and 8 replicates of each dilution analysed by real time PCR (Rotor Gene 6000, Corbett Life Science). The primer/probe sets were specific for the M9 MHV68 gene (primers M9-F 5’- GCCACGGTGGCCCTCTA and M9-R 5’–CAGGCCTCCCTCCCTTTG -3, and Taqman probe M9T, 5’- 6-FAM-CTTCTGTTGATCTTCC-MGB-3’). Positive and negative reactions were scored using Rotor Gene 6000 software and the frequency of infected cells was determined as described[46].

### Viral genome quantification

YFP^-^ and YFP^+^ GC B cells (CD19^+^GL7^+^CD95^+^) were FACS sorted from individual spleens of infected mice using a BD FACSAria Fow Cytometer (BD BioSciences). Viral genomes were detected by real time PCR using M9 primers and Taqman probe described above. Cellular DNA was quantifed in parallel by detecting the ribosomal protein L8 (Rpl8) gene (primers mRpl8 F1 5´-CATCCCTTTGGAGGTGGTA and mRpl8 R1 5´-CATCTCTTCGGATGGTGGA and Taqman probe mRpl8T 5´-VIC-ACCACCAGCACATTGGCAAACC-MGB) as previously described. Reactions were performed in a Rotor Gene 6000 (Corbett Life Science) and data were analysed with Rotor Gene 6000 software. PCR products were converted to genome copies by comparison to a standard curve of a plasmid harboring the MHV68 M9 gene or the rpl8 mouse gene (mouse cDNA NM_012053, Origene) serially diluted in the same buffer as the samples. The number of viral genenome copies per cell was obtained by dividing the number of M9 copies by one half the number of Rpl8 copies.

### Analysis of spleen sections

Spleens were fixed in 10% formalin in PBS for 24 hours at room temperature and paraffin embedded. In situ hybridization to detect MuHV-4-encoded miRNAs was performed in 5μm sections using digoxigenin-labelled riboprobes generated by T7 transcription of pEH1.4, as previously described[30]. For detection of LANA proteins, 4μm sections were cut, de-waxed, acid-treated for antigen recovery and blocked with Protein block (DAKO) for kLANA staining or using the Mouse on Mouse kit (DAKO) for mLANA staining. Sections were incubated for for 1 hour at room temperature with anti-mLANA mAb 6A3 (1:2 dilution of hybridoma supernatant) or rat anti-kLANA antibody (1:1000). For immunohistochemistry primary antibodies were detected with the anti-mouse ENVISION kit (DAKO) or ImmPress HRP anti-Rat IgG Peroxidase Polymer Detection Kit (Vector Laboratories), which use the Peroxidase/DAB detection system. Slides were counterstained with haematoxylin-eosin and images were acquired with a Leica DM2500 microscope coupled to a digital camera. For immunofluorescence, primary antibodies were detected with anti-mouse Alexa 594 or anti-rat Alexa-568 antibodies (Molecular Probes). Sections were mounted in Prolong gold reagent with DAPI (Molecular Probes) to stain DNA. Images were acquired with a Zeiss LSM 710 confocal microscope at 630x magnification and using Zen software. Images were processed with Image J. For quantification of dots, confocal z-stacks of nuclei, identified with DAPI staining, containing mLANA or kLANA dots were acquired from different areas of splenic follicles. The number of dots in individual nuclei was counted D across the z-stacks. Because most nuclei were not whole in the spleen sections, we determined the number of dots per nuclear volume. First we determined the nuclear volume in each slice, by multiplying the nuclei area, measured using ImageJ, by the thickness of the confocal slice. The final volume for each nucleus was the sum of the slice volumes.

### Statistical analysis

Statistical significance was evaluated with unpaired two-tailed non-parametric Mann-Whitney U test or one-way non-parametric ANOVA (Kruskal-Wallis test), as appropriate, using GraphPad Prism software.

## Supporting information

S1 Text TR episomes enlarge through recombination events. (DOCX)

S2 Text mLANA acts *in trans* on mTRs to mediate episome persistence. (DOCX)

S3 Text Reciprocal binding of kLANA and mLANA to TR DNA recognition sequences. (DOCX)

S4 Text Supporting information related materials and methods. (DOCX)

S1 Fig TR episomes enlarge through recombination events. (A) Schematic diagram showing digestion sites for m4TR, m8TR or pk8TR plasmids with NotI, or HindIII/XhoI, respectively. Sizes of expected fragments are indicated. TR, one mTR element; tr, one kTR element; us, unique KSHV sequence. (B, C) BJAB cell lines stably expressing kLANA transfected with pk8TR (Fig 1B, lanes 4, 5), pRepCK (Fig 1B, lanes 6 and 7), m8TR (Fig 1C, lanes 19 to 21), or m4TR (Fig 1B, lane 16), were harvested at 76 days of G418 selection. Low molecular weight DNA was isolated, digested with NotI (panel B) or HindIII and XhoI (panel C), and assessed by Southern blot. Exposure times are indicated below each blot. Lane letters correspond to the same letters as in Fig 1. Panel on the left in (B) is a 16 hour exposure and on the right shows lanes 6 to 11 after a 4 day exposure.
(JPG)

S2 Fig Rescued mTR plasmids from BJAB-kLANA cells have variable mTR copy number and can persist as episomes after transfection. After ~90 days of G418 selection, low molecular weight DNA was purified from a G418 resistant BJAB-kLANA cell line containing k8TR episomes (cell line a, shown in Fig 1C, lane 3, and in Fig 1D, lane 5) or from two G418 resistant BJAB-kLANA cell lines containing m8TR episomes (cell line d, shown in Fig 1C, lane 8 and Fig 1D, lane 10, and cell line f, shown in Fig 1C, lane 10 and Fig 1D, lane 11), transformed by electroporation into bacteria, and bacteria selected for ampicillin resistance. (A) Restriction enzyme digestion with NotI. (B) Restriction enzyme digestion with HindIII and XhoI. (C) Restriction enzyme digestion with HindIII. (D) Gardella gel analysis of Rm8TR-f.i in BJAB-kLANA cells after 58 days of G418 selection. Blot was probed with ^32^P-m8TR DNA. O, gel origin. Vertical line at right indicates mTR episomes. For plasmid DNA, the fastest migrating signal is circular, covalently closed DNA. Lane 13 was positive for episomal DNA on longer exposure. Smeared signal in lanes 12–23 in lower half of gel is due to DNA degradation.
(JPG)

S3 Fig mLANA mediates episome persistence in *trans*. (A) Immunoblots of mLANA detected by 6A3 monoclonal or FLAG antibody. A20 cells are G418 resistant. mLANAF-m4TR lanes are from cells with episomes at 47 days of G418 selection. Lane 9 has A20 cells transfected with mLANAF-m4TR at 3 days post transfection. mLANAF migrated slightly slower than S11 mLANA due to the 3xFLAG. 0.5x10^6^ cells (top panel) or 0.25x10^6^ cells (FLAG blot) were loaded per lane. (B, C) Gardella gel analyses of A20 cells or A20-mLANA cell lines transfected with m4TR-P. 2-3x10^6^ cells were loaded per lane. O, gel origin; E, S11 episomes; L, S11 linear genomes due to lytic replication; vertical lines indicate m4TR episomes; asterisk indicates ccc plasmid DNA. (D) Immunoblot of mLANA puromycin resistant cell lines from panel C. (E) Immunoblot of mLANA puromycin resistant cell lines from panel D. 0.25x10^6^ cells were loaded per lane. Bottom panels show tubulin.
(JPG)

S4 Fig Reciprocal binding of kLANA and mLANA to TR DNA. (A) Alignment of kLBS 1–2 with mLBS 1–2. Identical nucleotides are shown in blue. (B) EMSA with in vitro translated mLANA or kLANA or (C) EMSA with purified mLANA or kLANA DBD. (B) Long exposure of lanes 11–14 is shown at right. Star (lane 18) or asterisks (lane 14) indicate supershifted complexes. (C) solid circle indicates shifted complexes in lanes 13, 14. P, free ^32^P probe.
(TIF)

S5 Fig Lytic growth of MHV68 chimeric viruses. Expression of viral proteins after infection of BHK-21 cells for 6 hours with 3 PFU/cell of v-WT or v-kLANA virus. (A) Confocal images after immunofluorescence staining of mLANA or kLANA. DNA was stained with DAPI. Magnification 630x. (B) Immunoblot for kLANA, mLANA, vCyclin, or M3. (C) BHK-21 cells were infected with 0.01 PFU/cell of v-WT or v-kLANA virus and titers determined. There was no significant statistical difference between infection groups (p>0.05 using one-way non-parametric ANOVA Kruskal-Wallis.) (D) Lung virus titers after infection with 10^4^ PFU of the indicated viruses. Each circle represents an individual mouse, bars indicate the mean. Titers differed significantly between v-WT and v-Δ1007-21.yfp at day 7 (*p<0.05, using one-way non-parametric ANOVA Kruskal-Wallis followed by Dunn´s multiple comparison test). There were no other statistically significant differences.
(TIF)

S6 Fig v-kLANA latent infection after intranasal or intraperitoneal inoculation. C57 Bl/6 mice were infected with v-WT or v-kLANA virus and spleens were harvested at day 11, 14 (panels A, B) or 21 (C, D) after infection. (A, C) Reactivation virus titers (closed circles) and titers of pre-formed infectious virus (open circles). Circles represent titers of individual mice. Bars indicate mean and dashed line shows the limit of detection of the assay. (B, D) Frequency of infected splenocytes determined in parallel in pools of spleens from each infection group. Bars represent the frequency of viral DNA-positive cells and error bars indicate 95% confidence intervals.
(TIF)

S1 Table Puromycin or G418 resistant cell outgrowth. (DOCX)

S2 Table Oligonucleotides used for EMSAs. (DOCX)

S3 Table Detection of mTR episomes in BJAB-kLANA cells after transfection of plasmids containing greater than eight copies of mTR. (DOCX)
