# Supplementary material for: Cross-species conservation of episome maintenance provides a basis for in vivo investigation of Kaposi's sarcoma herpesvirus LANA
Source: PLoS Pathog. 2017 Sep 14;13(9):e1006555. doi: 10.1371/journal.ppat.1006555 (PMC5599060; doi:10.1371/journal.ppat.1006555)
Supplement: S1 Text — (DOCX) [file ppat.1006555.s001.docx]

**Supporting Information**

**S1 Text. TR episomes enlarge through recombination events**

Following growth under G418 selection, k8TR and mTR episomes migrated more slowly than input plasmid ccc DNA on Gardella gels, consistent with substantial enlargement through recombination events. After 27 days of G418 selection, most k8TR episomal DNA migrated similarly to ccc pk8TR plasmid DNA (Fig. 1B, compare predominant bands in lanes 3 and 4 with fastest migrating, ccc k8TR DNA band in lane 1). However, after 87 days of G418 selection, the episomal DNA migrated much more slowly than ccc pk8TR DNA (Fig. 1D, compare lanes 5, 6 with lane 1). Similarly, both m4TR and m8TR episomal DNA migrated much more slowly than ccc plasmid DNA (Fig. 1B-D, compare episomal DNA bands with fastest migrating, ccc m4TR (Fig. 1B, lane 3; Fig. 1D, lane 4) or m8TR (Fig. 1C, lane 2; Fig. 1D, lane 3) bands. We previously observed selection for larger episomes through recombination events as cells are carried in culture over time[[1-5](#_ENREF_1)].

To investigate potential recombination events, low molecular weight DNA was isolated, digested with NotI (S1B Fig.) or HindIII and XhoI (S1C Fig.), and assessed by Southern blot. NotI digestion of k8TR episomal DNA (S1B Fig.) resulted in expected ~6.2 kb vector size bands (S1B Fig., lanes 4, 5)(see S1A Fig. for expected sizes.) NotI digestion of mTR episomes resulted in the expected ~4.9 kb vector (arrow) and ~1.2 kb mTR (arrowhead) bands. The mTR band is hyperintense due to multiple mTR copies. Additional, bands migrating above or below vector band for cell lines o and p (S1B Fig., lanes 8, 9) represent likely aberrant recombination events.

HindIII/XhoI digestion linearizes k8TR plasmid to a ~12.6 kb band (S1C Fig., lane 1) and digestion of Hirt DNA from k8TR cell lines (S1C Fig., lanes 5, 6) had major bands of ~12.6 kb (although cell line b also had an additional, minor band.) Since HindIII/XhoI linearizes k8TR plasmid, this result suggests the large episomes are comprised of tandem head to tail k8TR multimers. HindIII/XhoI digestion of m4TR plasmid DNA produced a single band since the plasmid vector (~4.9kb) and insert of four mTR elements (~4.8 kb) co-migrate (S1C Fig., lane 4) and digestion of Hirt DNA from m4TR cell line i (S1C Fig., lane 12) also produced a ~4.9 kb band. HindIII/XhoI digestion of m8TR plasmid DNA produced a band at ~9.6 kb (from eight 1.2 kb mTR elements) and the 4.9kb plasmid vector backbone (S1C, lane 3). Digestion of Hirt DNA from m8TR cell lines had the expected ~9.6 kb mTR (arrowhead) and ~4.9 kb vector (arrow) bands (S1C Fig., lanes 9-12). m8TR cell line q (S1C Fig., lane 11) had one additional band of ~10.8kb, consistent with 9 mTR copies, and lines o and p (S1C Fig., lanes 9, 10) had additional bands that could result from expansion or contraction of mTR copy number and/or vector rearrangements. Overall, these results suggest the large, recombinant episomes are comprised of input plasmids arranged in tandem head to tail multimers, and that additional recombination events, likely including expansion and contraction of tandem mTRs also occur.

To further investigate recombinant events in mTR episomes persisting in BJAB-kLANA cells, episomal DNA clones were isolated in bacteria (S2A-C Fig.) After ~90 days of G418 selection, low molecular weight DNA was purified from a G418 resistant BJAB-kLANA cell line containing k8TR episomes (cell line a, shown in Fig. 1C, lane 3, and in Fig. 1D, lane 5) or from two G418 resistant BJAB-kLANA cell lines containing m8TR episomes (cell line d, shown in Fig. 1C, lane 8 and Fig. 1D, lane 10, and cell line f, shown in Fig. 1C, lane 10 and Fig. 1D, lane 11), transformed by electroporation into bacteria, and bacteria selected for ampicillin resistance. We only expected to rescue smaller episomes, since large episomes comprised of tandem multimers of ~14.5 kb input plasmid are unlikely to efficiently transform or persist in bacteria. Transformation of DNA from cells containing k8TR episomes yielded over 800 cfu, compared with six cfu for cell line “d” and eight cfu for cell line “f”. Rescue of only 6 and 8 m8TR clones compared with over 800 k8TR clones was likely due to fewer m8TR episomes, especially those of smaller size comigrating with ccc plasmid DNA, present in these cells, and consistent with the fainter signal in the Gardella gel analysis for m8TR DNA cell lines “d” and “f” compared with that of the k8TR cell line “a” (Fig. 1C, D).

Rescued m8TR (designated Rm8TR) plasmids were assessed by restriction enzyme analyses. Digestion with NotI, which releases mTR elements from the vector (S1A Fig.), resolved all but one plasmid (S2A, lane 1) into expected size vector (~4.9kb) and mTR (~1.2 kb) bands. Digestion with HindIII and XhoI, which releases the mTRs en bloc from the vector (S1A Fig.), revealed several clones with larger mTR cassettes (S2B, lanes 6, 7, 10-12) than the input m8TR plasmid (lane 13). The plasmid in S2B, lane 2 has four mTR copies resulting in co-migration of the mTR band with vector. The ~1.2kb size of the mTR band in S2B Fig., lane 8 was consistent with one mTR copy and the ~2.4kb band in lane 9 was consistent with two mTR copies. For one clone, HindIII/XhoI digestion resulted in an additional ~1.2kb mTR band of one mTR (S2B, Fig., lane 4), consistent with an erroneous recombination event. Digestion with HindIII, which linearizes the m8TR plasmid (S1A Fig.) showed plasmids contained a single band of expected size (when accounting for the increased or decreased number of mTR elements) except for two (S2C, lanes 1 and 4), each of which contained two bands, consistent with erroneous recombination events. Therefore, all but one of the rescued m8TR episomes contained recombination events within the mTR elements resulting in expansion or contraction of the number of mTRs.

To assess whether rescued m8TR plasmids containing more than eight m8TR copies were enhanced for episome persistence efficiency in BJAB-kLANA cells, k8TR, pRepCK vector, or rescued plasmid DNA derived from Rm8TR-f.i (S2 Fig, lane 6 in panels A, B, and C) or three other clones (S3 Table) was transfected into BJAB-kLANA cells, and cells were seeded into microtiter plates and placed under G418 selection. Gardella gel analysis was performed after 58 days of G418 selection. Only one rescued mTR clone resulted in a higher percentage of episome containing cell lines (S2D Fig., S3 Table) compared to that of the parental m8TR DNA (38%) (Table 1) indicating that the increased numbers of mTR elements in these plasmids did not consistently enhance episome maintenance efficiency.

**Supporting Information References**

1. Ballestas ME, Kaye KM (2001) Kaposi's sarcoma-associated herpesvirus latency-associated nuclear antigen 1 mediates episome persistence through cis-acting terminal repeat (TR) sequence and specifically binds TR DNA. J Virol 75: 3250-3258.

2. De Leon Vazquez E, Kaye KM (2011) The internal Kaposi's sarcoma-associated herpesvirus LANA regions exert a critical role on episome persistence. J Virol 85: 7622-7633.

3. De Leon Vazquez E, Carey VJ, Kaye KM (2013) Identification of Kshv Lana Regions Important for Episome Segregation, Replication and Persistence. J Virol 87: 12270-12283.

4. De Leon Vazquez E, Juillard F, Rosner B, Kaye KM (2014) A short sequence immediately upstream of the internal repeat elements is critical for KSHV LANA mediated DNA replication and impacts episome persistence. Virology 448: 344-355.

5. Habison AC, Beauchemin C, Simas JP, Usherwood EJ, Kaye KM (2012) Murine Gammaherpesvirus 68 LANA Acts on Terminal Repeat DNA To Mediate Episome Persistence. J Virol 86: 11863-11876.
