# Supplementary material for: Cross-species conservation of episome maintenance provides a basis for in vivo investigation of Kaposi's sarcoma herpesvirus LANA
Source: PLoS Pathog. 2017 Sep 14;13(9):e1006555. doi: 10.1371/journal.ppat.1006555 (PMC5599060; doi:10.1371/journal.ppat.1006555)
Supplement: S2 Text — (DOCX) [file ppat.1006555.s002.docx]

**Supporting Information**

**S2 Text. mLANA acts *in trans* on mTRs to mediate episome persistence.**

mLANA acts on mTRs to mediate episome persistence when both are *in cis* [[1](#_ENREF_1)], and we assessed here if mLANA can also act *in trans* to mediate persistence. For these experiments, we used two independently derived G418 resistant cell lines, A20-mLANAF(A) or A20-mLANAF(B), each of which stably express mLANAF. A20-mLANAF(B) (S3A Fig.) expressed mLANA at a slightly reduced level compared to S11, an MHV68 latently infected, murine B tumor cell line, while mLANA expression was lower in A20-mLANAF(A) (described in [[1](#_ENREF_1)]) (S3A Fig.). However, mLANA levels in A20-mLANAF(A) and (B) were similar to or higher than that of mLANA in two cell lines stably maintaining episomes containing mLANA and four mTRs *in cis* (mLANAF—m4TR) (described in [[1](#_ENREF_1)]) (S3A Fig.). Therefore, A20-mLANAF(A) and (B) express mLANA at physiologic levels. A20-mLANAF(A) and (B) grew more slowly than parental A20 cells, consistent with a mild mLANA inhibitory effect on cell growth as previously observed [[1](#_ENREF_1)]; growth rates inversely correlated with mLANA expression levels with A20-mLANAF(B), doubling at about half the rate of A20 cells.

DNA containing four mTR copies (m4TR-P) or vector (pRepCK-P) was transfected into A20-mLANAF(A) or A20-mLANAF(B) cells. Puromycin resistant outgrowth (conferred by the plasmid vector) was low after transfection of pRepCK-P and higher after transfection of m4TR-P regardless of the presence of mLANA (S1 Table). This result could be due to absence of mLANA episome persistence or episome persistence efficiency similar to that of integration. In the absence of LANA episome maintenance, integration is required for m4TR persistence. m4TR DNA integrated in A20 cells at higher rates than pRepCK-P vector, similar to previous observations (S1 Table) [[1](#_ENREF_1)].

After expansion of cells, Gardella gel analyses[[2](#_ENREF_2)] were performed to assess for the presence of episomes. As expected, no episomes were present in the absence of mLANA (S3B Fig.). In contrast, m4TR-P episomes were present in 10 of 15 lanes for A20-mLANAF (B) (S3B Fig.). Most episomal DNA migrated much more slowly than the circular, covalently closed (ccc) plasmid (S3B Fig., asterisk), migrating similarly to ~200kb MHV68 episomes (E) in the S11 cells. In five experiments with A20-mLANAF(B) cells, 48 of 83 (58%) of puromycin resistant cell lines had episomes (Table 1). Episomal DNA was also present in one of six puromycin resistant A20-mLANAF(A) cell lines (S3C Fig.). In two experiments with A20-mLANAF(A) cells, 5 of 20 (25%) cell lines had episomes. The lower percentage of puromycin resistant cell lines with m4TR-P episomes may be due to the lower mLANA expression level in the A20-mLANAF(A) cells compared with that of A20-mLANAF(B) (S3A Fig.). In contrast, episomes were never seen in 34 puromycin resistant cell lines after transfection of m4TR-P into A20 cells. Immunoblot analysis demonstrated that puromycin resistant A20-mLANAF(A) or (B) cells) continued to express mLANAF, even in cell lines that lacked episomes (S3D, S3E Fig.). Consequently, the absence of episomes in some puromycin resistant cell lines was not due to lack of mLANA expression. Therefore, mLANA acts in trans on mTR DNA to mediate episome persistence.

**Supporting Information References**

1. Habison AC, Beauchemin C, Simas JP, Usherwood EJ, Kaye KM (2012) Murine Gammaherpesvirus 68 LANA Acts on Terminal Repeat DNA To Mediate Episome Persistence. J Virol 86: 11863-11876.

2. Gardella T, Medveczky P, Sairenji T, mulder C (1984) Detection of circular and linear herpesvirus DNA molecules in mammalian cells by gel electrophoresis. Journal of Virology 50: 248-254.
