# Supplementary material for: Cross-species conservation of episome maintenance provides a basis for in vivo investigation of Kaposi's sarcoma herpesvirus LANA
Source: PLoS Pathog. 2017 Sep 14;13(9):e1006555. doi: 10.1371/journal.ppat.1006555 (PMC5599060; doi:10.1371/journal.ppat.1006555)
Supplement: S4 Text — (DOCX) [file ppat.1006555.s004.docx]

**Supporting Information**

**S4 Text. Materials and Methods.**

**Hirt DNA extraction, digestion and Southern blot.** Ten million G418 resistant BJAB-kLANA cells transfected with m4TR, m8TR, k8TR, or vector control (pRepCK) were harvested after 76 days of selection for low molecular weight DNA isolation by the Hirt method[[1](#_ENREF_1)]. Cells were lysed in 2 ml lysis buffer (0.6%SDS, 10mM EDTA, 10mM Tris-HCl pH 7.5, 50ug/ml RnaseA) and incubated at 37°C for 2 hours. NaCl was then added to a final concentration of 1M and incubated overnight at 4°C. After centrifugation at 11,000 x g at 4°C for 30 minutes to precipitate chromosomal DNA, low molecular weight DNA was extracted with phenol:chloform (1:1), then twice with chloroform:isoamyl alcohol (24:1) and ethanol precipitated. The DNA pellet was washed with 70% ethanol, air dried and resuspended in TE buffer (10mM Tris pH8, 0.1mM EDTA). 30µg of Hirt DNA was digested overnight with either NotI or HindIII and XhoI, resolved in a 0.8% agarose gel and transferred to a nylon membrane for detection by Southern blotting with ^32^P radiolabeled probe.

**Plasmid rescue of episomal DNA into bacteria.** Low molecular weight DNA was isolated from ten million G418 resistant BJAB-kLANA cells initially transfected with pk8TR, m4TR, m8TR or pRepCK, using Qiagen’s R.E.A.L. Prep 96 Plasmid Kit according to the manufacturer’s instructions, with the exception that 2x volumes of R1, R2 and R3 were used and DNA pellets were resuspended in ddH_2_O. Ten µL of DH10B competent cells (New England Biolabs) were transformed by electroporation of 5µl of purified DNA using settings of 2.0 kV, 200 Ω, and 25 μF with a BTX *E. coli* TransPorator, plated onto LB-Ampicillin, and incubated at 30°C. For larger scale plasmid preparations, plasmids were transformed by heat shock into STBL2 competent cells (MaxiPrep Invitrogen) and DNA used for Southern blot analysis or transfection into BJAB-kLANA cells to assess episome maintenance.

**EMSA** For electrophoretic mobility shift assays (EMSA), T7-kLANA or Myc-mLANA-C3F were in vitro translated using TNT Quick-coupled reticulocyte lysate systems (Promega). Ten μL (or 20 µL) of in vitro-translated proteins were incubated in DNA binding buffer [20 mM Tris (pH 7.5), 10% glycerol, 50 mM KCl, 0.1 mM dithiothreitol, 10 mM MgCl_2_, 1 mM EDTA, 20 μg/ml of poly(dI-dC)] with 50,000 counts per minute of ^32^P-labeled TR probe for 30 min on ice. Probes were prepared by annealing kLBS (kLANA binding site) or mLBS encoding oligonucleotides (shown in S2 Table), and filling in the ends. For supershift assays, after the 30-min incubation, samples were incubated for 15 min at room temperature with 1 μg of anti-T7 tag antibody (Novagen) for kLANA or 1 μg of anti-Flag (Sigma) for mLANA. Fifty fold excess, unlabeled oligonucleotide was included for competition assays. Bound complexes were resolved on a 4% nondenaturing polyacrylamide gel. Signal was detected by autoradiography.

For EMSAs performed with purified protein, mLANA (mLANA_124-314_) or kLANA (kLANA_1008-1150_) proteins were expressed and purified as previously described[[2](#_ENREF_2),[3](#_ENREF_3)]. DNA probes corresponding to regions containing kLBS1-2 or mLBS1-2 were generated using fluorescein-labeled DNA oligonucleotides (Sigma-Aldrich), Flc-5’-TTT-mLBS1-2, Flc-5’-TTT-kLBS1-2, or unlabeled competitor oligonucleotide, kLBS1-2 (S2 Table). To generate probes, synthetic oligonucleotides were mixed together in equimolar amounts in buffer containing 10 mM Tris-HCl pH 8, 50mM NaCl, 5mM MgCl_2_ 1mM EDTA and annealed by heating the mixed oligonucleotides to 95°C for 10 minutes and gradually cooled in a stepwise manner to 4°C over a period of 2 h using a MyCycler™ thermal cycler (BioRAD). The annealed oligonucleotides were stored at −20°C prior to use.

Prior to incubation with oligonucleotides, proteins were buffer exchanged into buffer containing 20mM Tris-HCl pH7.5, 300mM KCl, 10mM MgCl_2_ and 10% glycerol. Binding reactions were performed overnight by incubating LANA protein with 1 µM of each DNA oligonucleotide, supplemented with 1mM EDTA, 0.01 mg/ml poly dI-dC, 0.1 mM DTT and 0.1% Tween 20, in a final volume of 30 µL. For supershift assays, five fold excess of purified α-mLANA 6A3 antibody was included in the incubation with the mLANA protein. For competition experiments, ten or fifty fold excess unlabeled oligonucleotide of kLBS1-2 was included in the incubation. Reactions were loaded on a native 4% TBE-acrylamide gel and run at 200V for 3h in 1X TBE buffer. Complexes were visualized after exposure to a Fujifilm-FLA5100 scanner with an LPB filter at 473 nm wavelength.

**Supporting Information References**

1. Hirt B (1967) Selective extraction of polyoma DNA from infected mouse cell cultures. J Mol Biol 26: 365-369.

2. Correia B, Cerqueira SA, Beauchemin C, Pires de Miranda M, Li S, et al. (2013) Crystal Structure of the Gamma-2 Herpesvirus LANA DNA Binding Domain Identifies Charged Surface Residues Which Impact Viral Latency. PLoS Pathog 9: e1003673.

3. Ponnusamy R, Petoukhov MV, Correia B, Custodio TF, Juillard F, et al. (2015) KSHV but not MHV-68 LANA induces a strong bend upon binding to terminal repeat viral DNA. Nucleic Acids Res 43: 10039-10054.
