# Supplementary figures and images for: Cross-species conservation of episome maintenance provides a basis for in vivo investigation of Kaposi's sarcoma herpesvirus LANA
Source: PLoS Pathog. 2017 Sep 14;13(9):e1006555. doi: 10.1371/journal.ppat.1006555 (PMC5599060; doi:10.1371/journal.ppat.1006555)

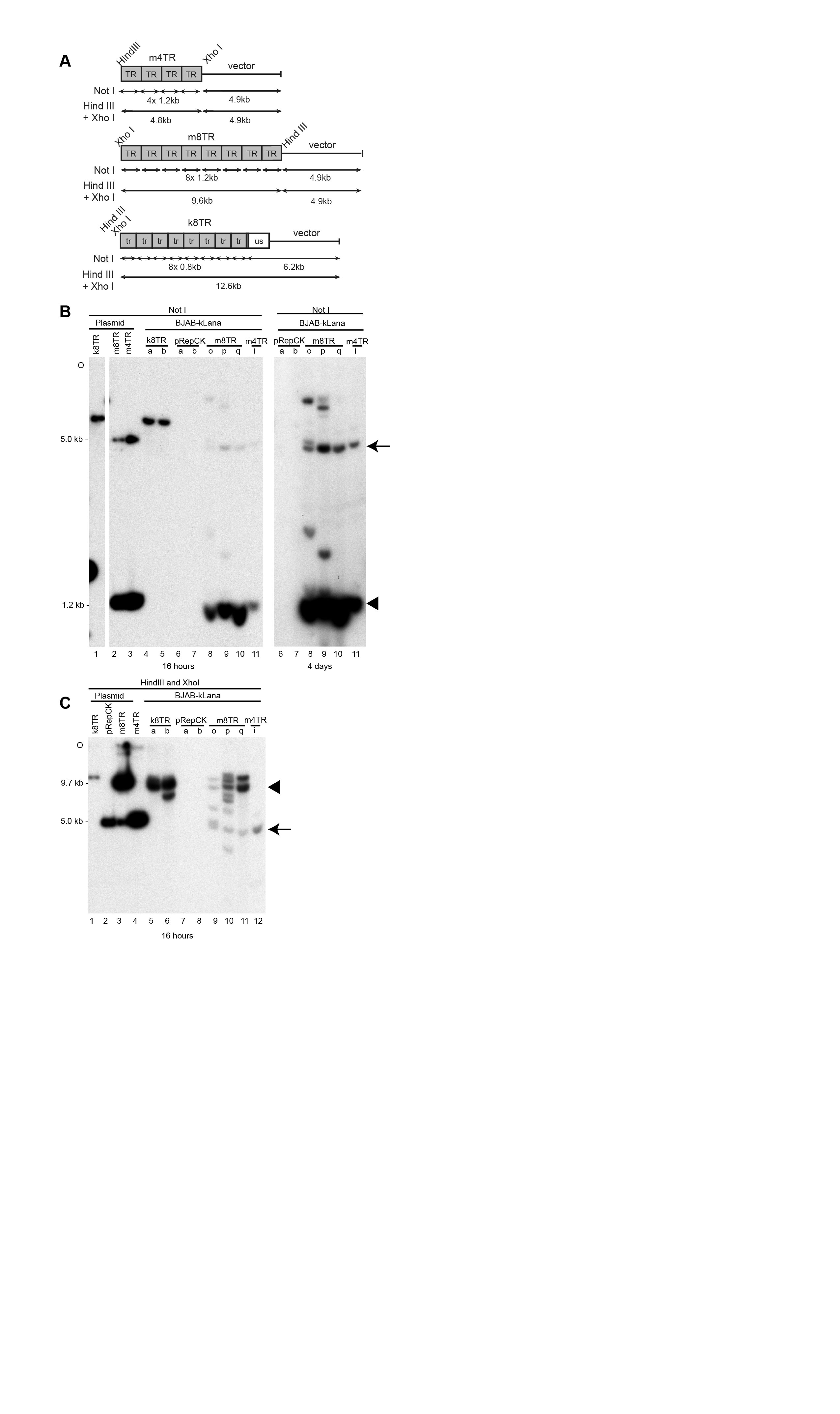

Supplement: S1 Fig — (A) Schematic diagram showing digestion sites for m4TR, m8TR or pk8TR plasmids with NotI, or HindIII/XhoI, respectively. Sizes of expected fragments are indicated. TR, one mTR element; tr, one kTR element; us, unique KSHV sequence. (B, C) BJAB cell lines stably expressing kLANA transfected with pk8TR (Fig 1B, lanes 4, 5), pRepCK (Fig 1B, lanes 6 and 7), m8TR (Fig 1C, lanes 19 to 21), or m4TR (Fig 1B, lane 16), were harvested at 76 days of G418 selection. Low molecular weight DNA was isolated, digested with NotI (panel B) or HindIII and XhoI (panel C), and assessed by Southern blot. Exposure times are indicated below each blot. Lane letters correspond to the same letters as in Fig 1. Panel on the left in (B) is a 16 hour exposure and on the right shows lanes 6 to 11 after a 4 day exposure. (JPG) [file ppat.1006555.s005.jpg]

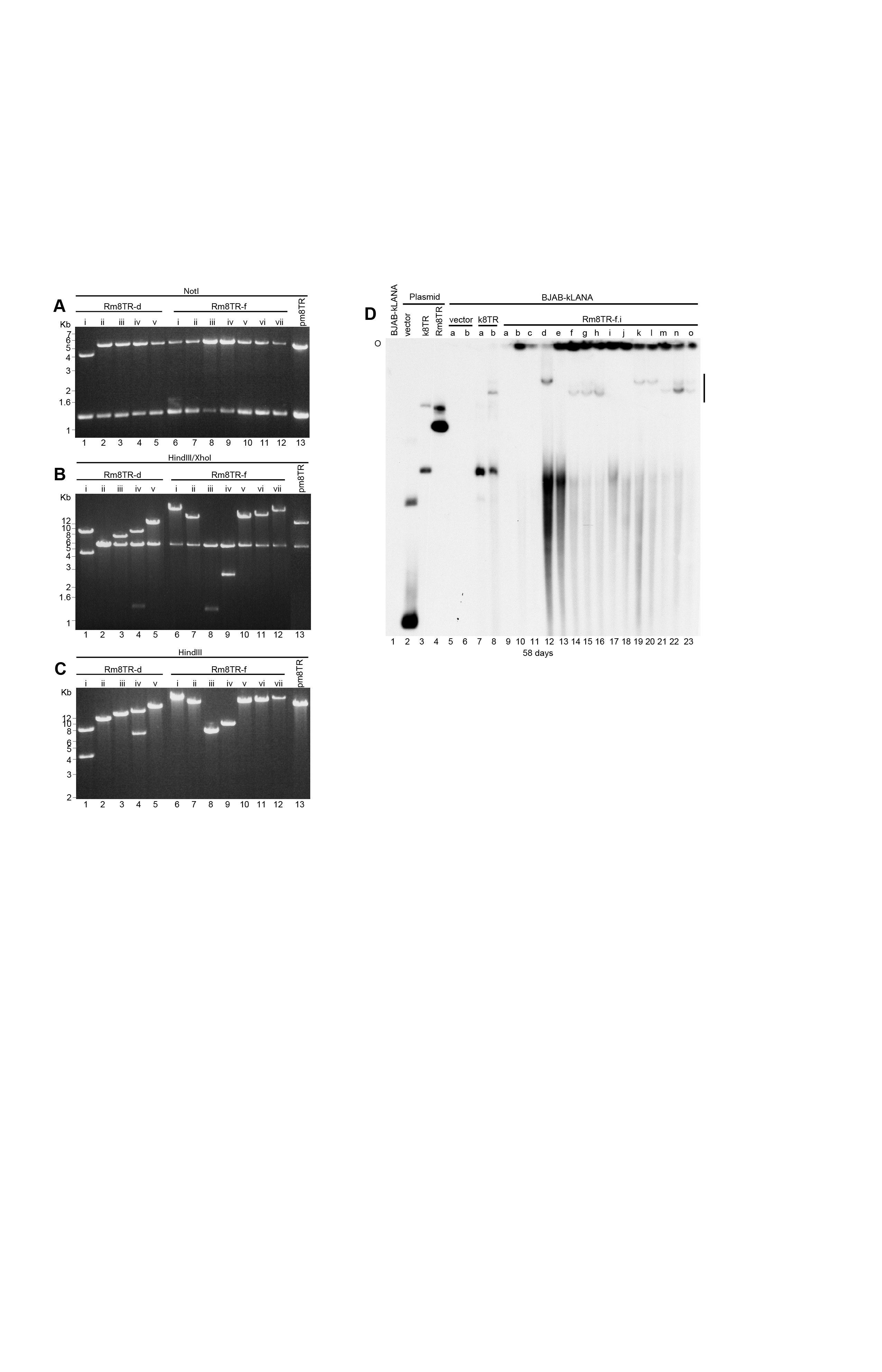

Supplement: S2 Fig — After ~90 days of G418 selection, low molecular weight DNA was purified from a G418 resistant BJAB-kLANA cell line containing k8TR episomes (cell line a, shown in Fig 1C, lane 3, and in Fig 1D, lane 5) or from two G418 resistant BJAB-kLANA cell lines containing m8TR episomes (cell line d, shown in Fig 1C, lane 8 and Fig 1D, lane 10, and cell line f, shown in Fig 1C, lane 10 and Fig 1D, lane 11), transformed by electroporation into bacteria, and bacteria selected for ampicillin resistance. (A) Restriction enzyme digestion with NotI. (B) Restriction enzyme digestion with HindIII and XhoI. (C) Restriction enzyme digestion with HindIII. (D) Gardella gel analysis of Rm8TR-f.i in BJAB-kLANA cells after 58 days of G418 selection. Blot was probed with 32P-m8TR DNA. O, gel origin. Vertical line at right indicates mTR episomes. For plasmid DNA, the fastest migrating signal is circular, covalently closed DNA. Lane 13 was positive for episomal DNA on longer exposure. Smeared signal in lanes 12–23 in lower half of gel is due to DNA degradation. (JPG) [file ppat.1006555.s006.jpg]

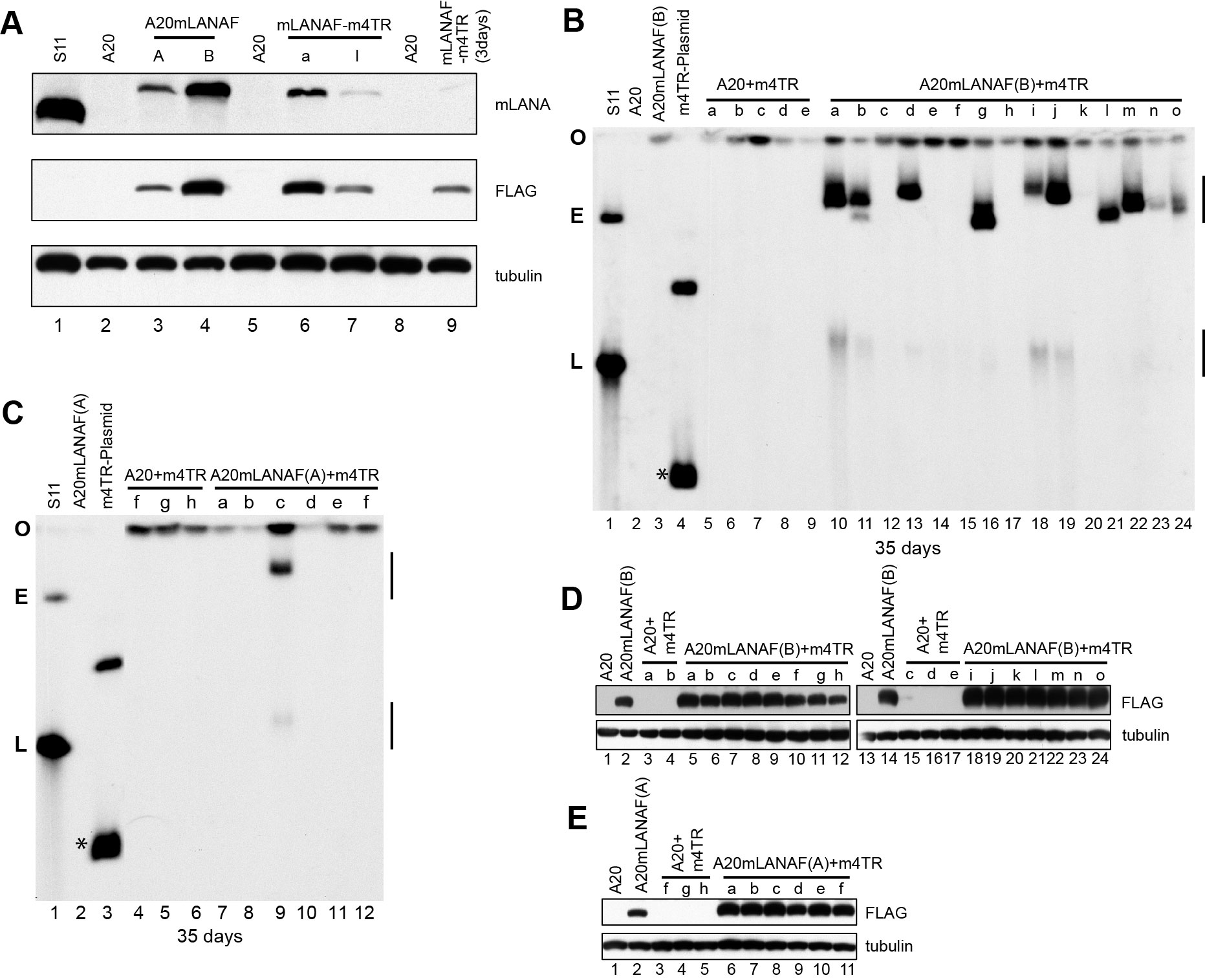

Supplement: S3 Fig — (A) Immunoblots of mLANA detected by 6A3 monoclonal or FLAG antibody. A20 cells are G418 resistant. mLANAF-m4TR lanes are from cells with episomes at 47 days of G418 selection. Lane 9 has A20 cells transfected with mLANAF-m4TR at 3 days post transfection. mLANAF migrated slightly slower than S11 mLANA due to the 3xFLAG. 0.5x10^6^ cells (top panel) or 0.25x10^6^ cells (FLAG blot) were loaded per lane. (B, C) Gardella gel analyses of A20 cells or A20-mLANA cell lines transfected with m4TR-P. 2-3x10^6^ cells were loaded per lane. O, gel origin; E, S11 episomes; L, S11 linear genomes due to lytic replication; vertical lines indicate m4TR episomes; asterisk indicates ccc plasmid DNA. (D) Immunoblot of mLANA puromycin resistant cell lines from panel C. (E) Immunoblot of mLANA puromycin resistant cell lines from panel D. 0.25x10^6^ cells were loaded per lane. Bottom panels show tubulin. (JPG) [file ppat.1006555.s007.jpg]

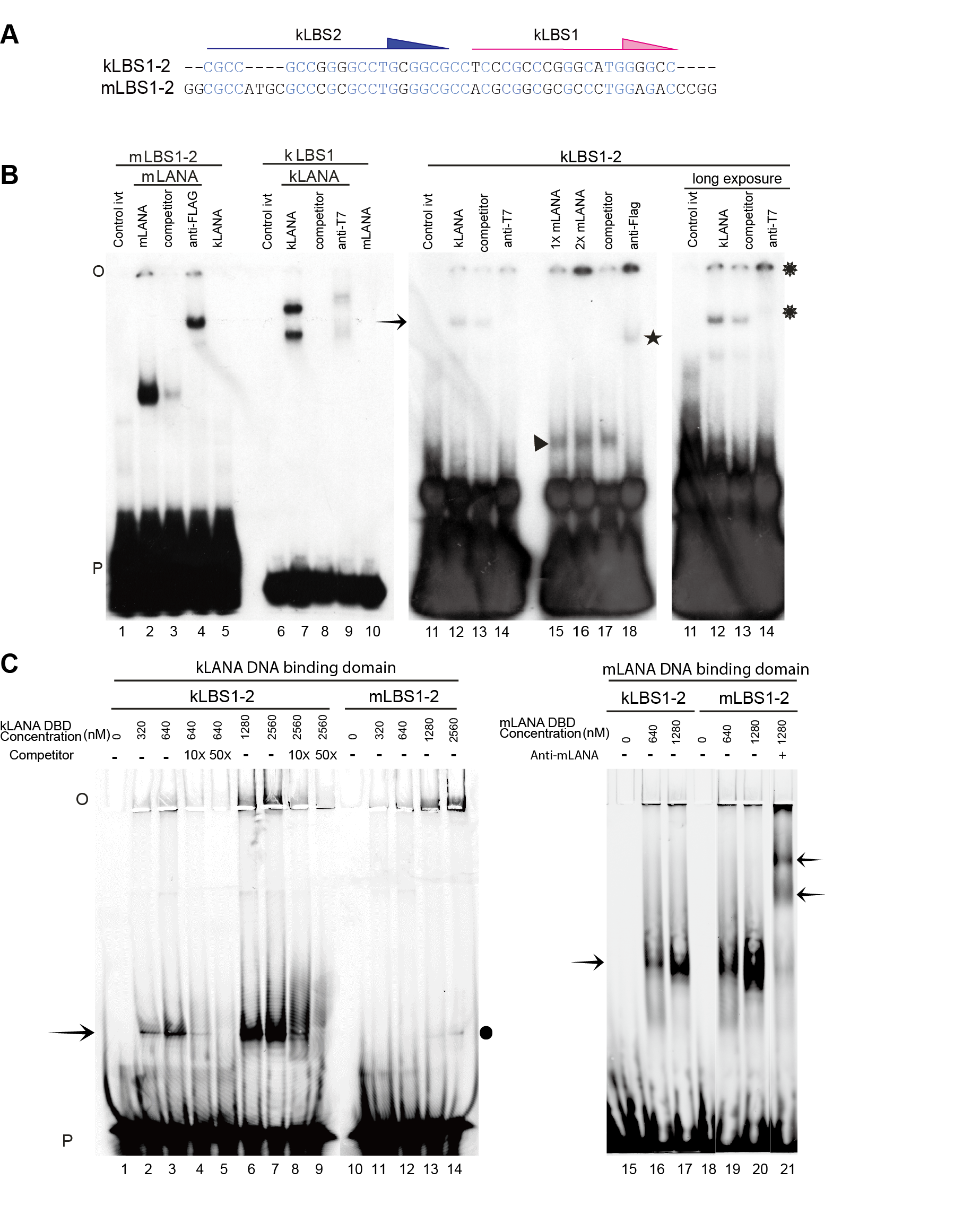

Supplement: S4 Fig — (A) Alignment of kLBS 1–2 with mLBS 1–2. Identical nucleotides are shown in blue. (B) EMSA with in vitro translated mLANA or kLANA or (C) EMSA with purified mLANA or kLANA DBD. (B) Long exposure of lanes 11–14 is shown at right. Star (lane 18) or asterisks (lane 14) indicate supershifted complexes. (C) solid circle indicates shifted complexes in lanes 13, 14. P, free 32P probe. (TIF) [file ppat.1006555.s008.tif]

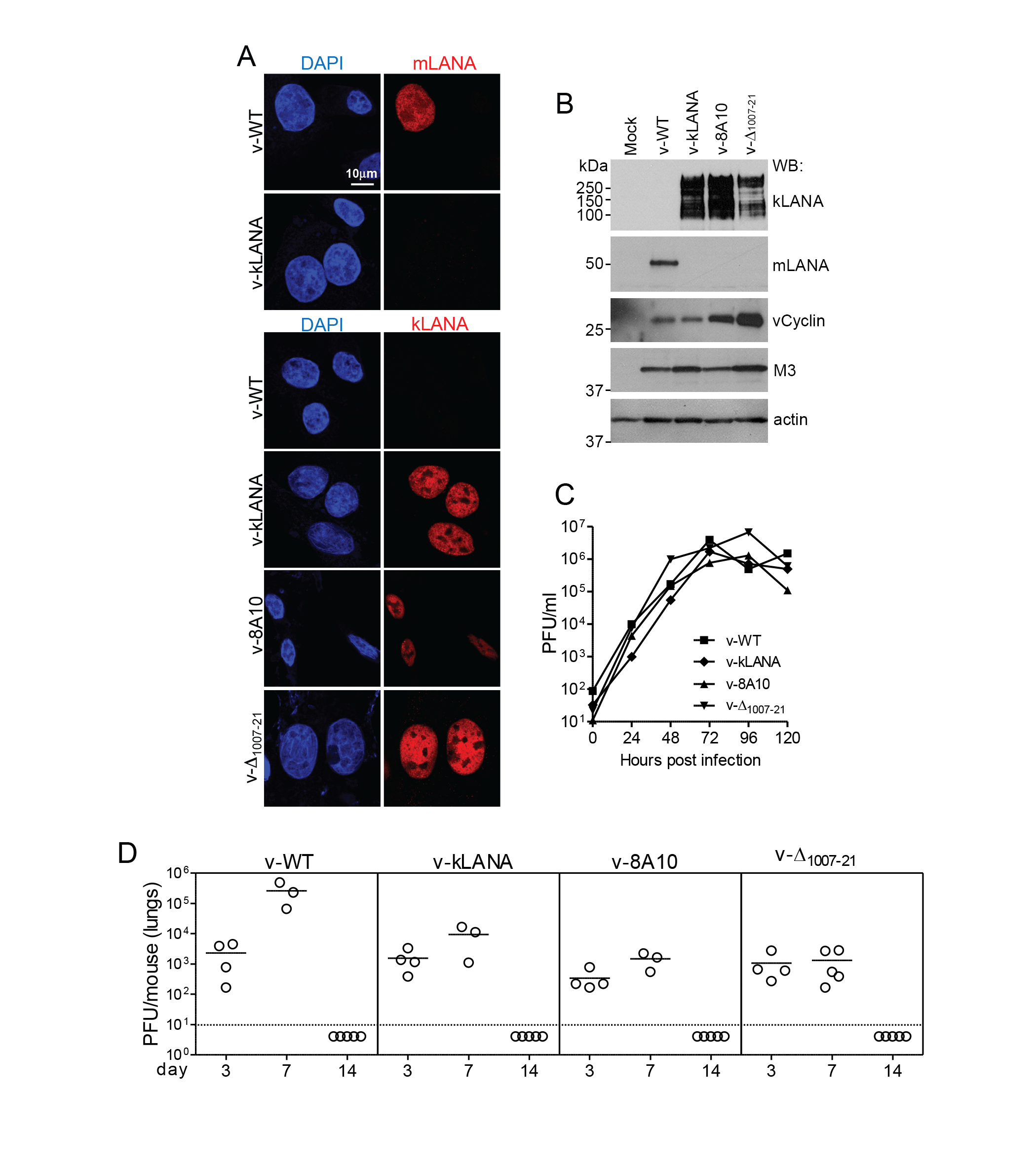

Supplement: S5 Fig — Expression of viral proteins after infection of BHK-21 cells for 6 hours with 3 PFU/cell of v-WT or v-kLANA virus. (A) Confocal images after immunofluorescence staining of mLANA or kLANA. DNA was stained with DAPI. Magnification 630x. (B) Immunoblot for kLANA, mLANA, vCyclin, or M3. (C) BHK-21 cells were infected with 0.01 PFU/cell of v-WT or v-kLANA virus and titers determined. There was no significant statistical difference between infection groups (p>0.05 using one-way non-parametric ANOVA Kruskal-Wallis.) (D) Lung virus titers after infection with 104 PFU of the indicated viruses. Each circle represents an individual mouse, bars indicate the mean. Titers differed significantly between v-WT and v-Δ1007-21.yfp at day 7 (*p<0.05, using one-way non-parametric ANOVA Kruskal-Wallis followed by Dunn´s multiple comparison test). There were no other statistically significant differences. (TIF) [file ppat.1006555.s009.tif]

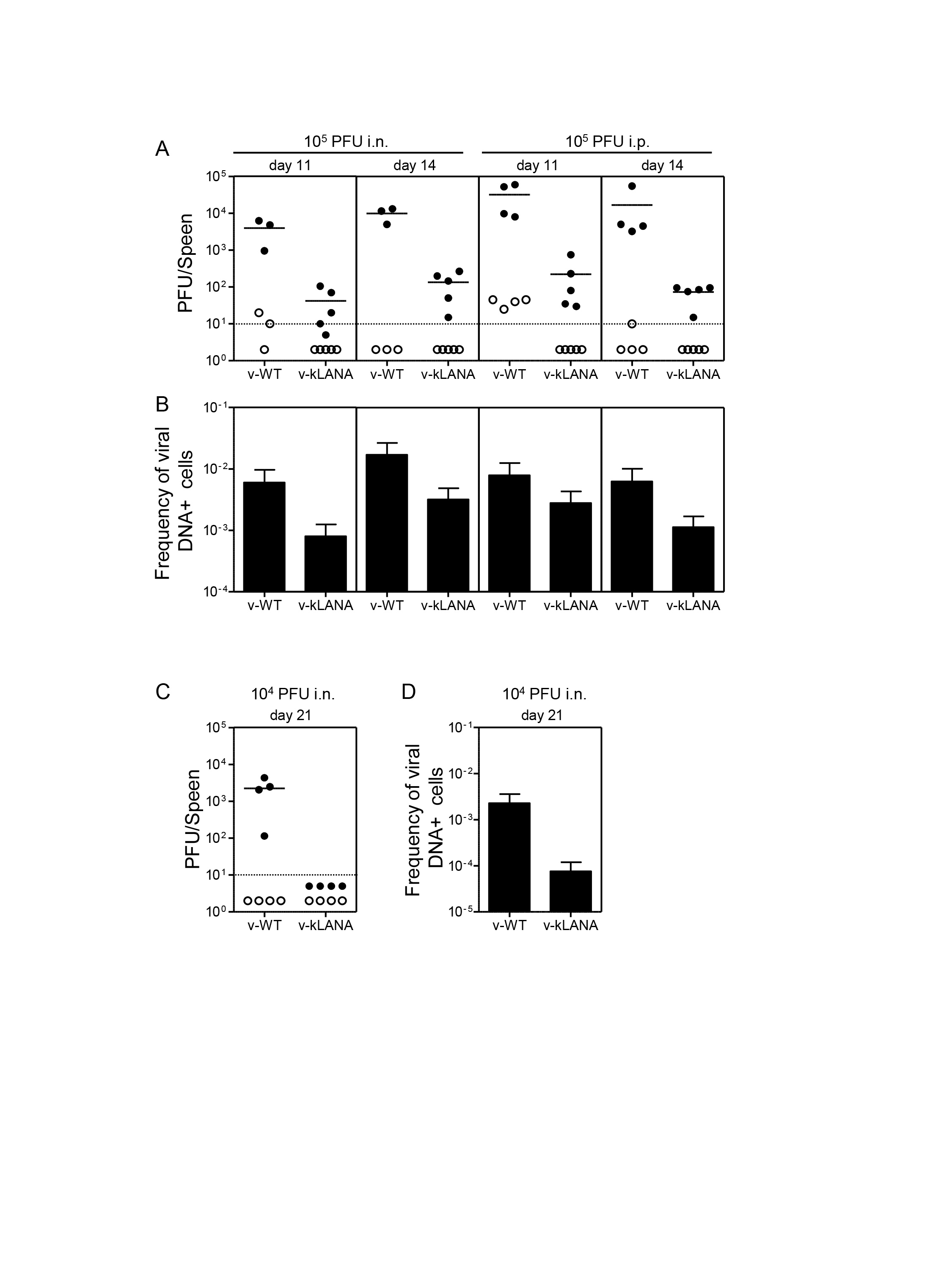

Supplement: S6 Fig — C57 Bl/6 mice were infected with v-WT or v-kLANA virus and spleens were harvested at day 11, 14 (panels A, B) or 21 (C, D) after infection. (A, C) Reactivation virus titers (closed circles) and titers of pre-formed infectious virus (open circles). Circles represent titers of individual mice. Bars indicate mean and dashed line shows the limit of detection of the assay. (B, D) Frequency of infected splenocytes determined in parallel in pools of spleens from each infection group. Bars represent the frequency of viral DNA-positive cells and error bars indicate 95% confidence intervals. (TIF) [file ppat.1006555.s010.tif]
