# Supplementary material for: Cross-species conservation of episome maintenance provides a basis for in vivo investigation of Kaposi's sarcoma herpesvirus LANA
Source: PLoS Pathog. 2017 Sep 14;13(9):e1006555. doi: 10.1371/journal.ppat.1006555 (PMC5599060; doi:10.1371/journal.ppat.1006555)
Supplement: S1 Table — (DOCX) [file ppat.1006555.s011.docx]

S1 Table. Puromycin or G418 resistant cell outgrowth

| Cell line | Transfected DNA^1^ | Outgrowth^2^  1000 cells/well | Outgrowth  100 cells/well | Outgrowth  10 cells/well |
| --- | --- | --- | --- | --- |
| BJAB-kLANA^3^ | pk8TR | 96 *(0)* | 96 *(0.4)* | 55 *(24)* |
| BJAB-kLANA^3^ | m2TR | 96 *(0.5)* | 41 *(9)* | 3 *(2)* |
| BJAB-kLANA^3^ | m4TR | 96 *(0.4)* | 85 *(3)* | 13 *(3)* |
| BJAB-kLANA^3^ | m8TR | 96 *(0)* | 94 *(3)* | 23 (*6)* |
| BJAB-kLANA^3^ | pRepCK | 55 *(18)* | 10 (*3)* | 1 (*0.5)* |
|  |  |  |  |  |
| BJAB^4^ | pk8TR | 62 (57,67) | 13 (8, 17) | 2 (1, 3) |
| BJAB^4^ | pRepCK | 50 (47, 52) | 6 (2, 9) | 1 (0, 1) |
| BJAB^4^ | m2TR | 77 (71, 82) | 14 (11, 16) | 2 (0, 3) |
| BJAB^4^ | m4TR | 84 (92, 76) | 23 (26, 20) | 3 (2, 3) |
| BJAB^4^ | m8TR | 95 (95, 95) | 40 (38, 41) | 10 (8, 12) |
|  |  |  |  |  |
| A20-mLANAF(A) | m4TR-P | 82 | 17 | 12 |
| A20-mLANAF(B) ^5^ | m4TR-P | 95 *(1)* | 23 *(5)* | 3 (*2)* |
| A20-mLANAF(A) | pRepCK-P | 1 | 0 | 0 |
| A20-mLANAF(B)^5^ | pRepCK-P | 9 *(5)* | 1 *(0.5)* | 0 *(0)* |
| A20^5^ | m4TR-P | 71 *(9)* | 18 (*6)* | 3 *(0.5)* |
|  |  |  |  |  |
| A20-mLANAF(B) ^4^ | pk8TR-P | 96 (96, 96) | 35 (23, 46) | 3 (3, 3) |
| A20^5^ | pk8TR-P | 43 *(19)* | 7 *(3)* | 1 *(0.5)* |
| A20^5^ | pRepCK-P | 13 *(8)* | 2 *(1)* | 0 *(0)* |

^1^Plasmids with “P” suffix encode puromycin resistance; others encode G418 resistance. ^2^Number of wells of puromycin or G418 resistant outgrowth per 96 well microtiter plate at the indicated seeding density. ^3^Average of at least three experiments with standard deviation shown in italics. ^4^Average of two experiments with individual values in parenthesis. ^5^Average of three experiments with standard deviation shown in italics.
