# Supplementary material for: Cross-species conservation of episome maintenance provides a basis for in vivo investigation of Kaposi's sarcoma herpesvirus LANA
Source: PLoS Pathog. 2017 Sep 14;13(9):e1006555. doi: 10.1371/journal.ppat.1006555 (PMC5599060; doi:10.1371/journal.ppat.1006555)
Supplement: S2 Table — (DOCX) [file ppat.1006555.s012.docx]

S2 Table. Oligonucleotides used for EMSAs^1^

| **LBS probe** | **dsDNA sequence** |
| --- | --- |
| kLBS1 | 5’- GGAT**TCCCGCCCGGGCATGGGGCC**G  3’- **AGGGCGGGCCCGTACCCCGG**CTAGG -5’ |
| kLBS1-2 | 5’-GATC***cgccgccggggcctgcggcg***cc**tcccgcccgggcatggggcc**g-3’  3’-***GCGGCGGCCCCGGACGCCGC***GG**AGGGCGGGCCCGTACCCCGG**CCTAG-5’ |
| mLBS1-2 | 5’- GATC**GGCGCCATGCGCCCGCGCCTGGGGCGCCACGCGGCGCGCCCTGGAGACCCGG**GG-3’  3’- **CCGCGGTACGCGGGCGCGGACCCCGCGGTGCGCCGCGCGGGACCTCTGGGCC**CCCTAG-5’ |
| Flc-kLBS1-2 | [Flc]5'-TTTGA***CGCCGCCGGGGCCTGCGGCG***CC**TCCCGCCCGGGCATGGGGCC**GC-3'  3'-CT***GCGGCGGCCCCGGACGCCGC***GG**AGGGCGGGCCCGTACCCCGG**CG-5' |
| Flc-mLBS1-2 | [Flc]5'-TTTCCCGCGCCTGGGGCGCCACGCGGCGCGCCCTGGAGACccggG-3'  3'-GGGCGCGGACCCCGCGGTGCGCCGCGCGGGACCTCTGGGCCC-5' |
| Unlabeled, competitor kLBS1-2 | 5'-GA***CGCCGCCGGGGCCTGCGGCG***CC**TCCCGCCCGGGCATGGGGCC**GC-3'  3'-CT***GCGGCGGCCCCGGACGCCGC***GG**AGGGCGGGCCCGTACCCCGG**CG |

^1^kLBS1[[1](#_ENREF_1)] is in bold and underlined, kLBS2 site is bold and italicized, mLBS1-2 is underlined[[2](#_ENREF_2),[3](#_ENREF_3)].

**Supporting Information References**

1. Garber AC, Hu J, Renne R (2002) Latency-associated nuclear antigen (LANA) cooperatively binds to two sites within the terminal repeat, and both sites contribute to the ability of LANA to suppress transcription and to facilitate DNA replication. J Biol Chem 277: 27401-27411.

2. Paden CR, Forrest JC, Tibbetts SA, Speck SH (2012) Unbiased Mutagenesis of MHV68 LANA Reveals a DNA-Binding Domain Required for LANA Function In Vitro and In Vivo. PLoS Pathog 8: e1002906.

3. Correia B, Cerqueira SA, Beauchemin C, Pires de Miranda M, Li S, et al. (2013) Crystal Structure of the Gamma-2 Herpesvirus LANA DNA Binding Domain Identifies Charged Surface Residues Which Impact Viral Latency. PLoS Pathog 9: e1003673.
