# Supplementary material for: Cross-species conservation of episome maintenance provides a basis for in vivo investigation of Kaposi's sarcoma herpesvirus LANA
Source: PLoS Pathog. 2017 Sep 14;13(9):e1006555. doi: 10.1371/journal.ppat.1006555 (PMC5599060; doi:10.1371/journal.ppat.1006555)
Supplement: S3 Table — (DOCX) [file ppat.1006555.s013.docx]

S3 Table. Detection of mTR episomes in BJAB-kLANA cells after transfection of plasmids containing greater than eight copies of mTR.

| Transfected DNA | Number of cell lines assessed | Cell lines with episomal DNA | Percent with episomal DNA |
| --- | --- | --- | --- |
| pRepCK | 3 | 0 | 0 (%) |
| pk8TR | 2 | 2 | 100 (%) |
| Rm8TR-f.i | 15 | 10 | 67 (%) |
| Rm8TR-f.v | 15 | 5 | 33 (%) |
| Rm8TR-f.vi | 15 | 5 | 33 (%) |
| Rm8TR-f.vii | 15 | 1 | 7 (%) |
